# RNA-seq-based comparative transcriptome analysis reveals the role of *CsPrx73* in waterlogging-triggered adventitious root formation in cucumber

**DOI:** 10.1093/hr/uhae062

**Published:** 2024-02-28

**Authors:** Jiawei Pan, Jia Song, Hamza Sohail, Rahat Sharif, Wenjing Yan, Qiming Hu, Xiaohua Qi, Xiaodong Yang, Xuewen Xu, Xuehao Chen

**Affiliations:** Department of Horticulture, School of Horticulture and Landscape Architecture, Yangzhou University, Yangzhou, Jiangsu 225009, China; Department of Horticulture, School of Horticulture and Landscape Architecture, Yangzhou University, Yangzhou, Jiangsu 225009, China; Department of Horticulture, School of Horticulture and Landscape Architecture, Yangzhou University, Yangzhou, Jiangsu 225009, China; Department of Horticulture, School of Horticulture and Landscape Architecture, Yangzhou University, Yangzhou, Jiangsu 225009, China; Department of Horticulture, School of Horticulture and Landscape Architecture, Yangzhou University, Yangzhou, Jiangsu 225009, China; Department of Horticulture, School of Horticulture and Landscape Architecture, Yangzhou University, Yangzhou, Jiangsu 225009, China; Department of Horticulture, School of Horticulture and Landscape Architecture, Yangzhou University, Yangzhou, Jiangsu 225009, China; Department of Horticulture, School of Horticulture and Landscape Architecture, Yangzhou University, Yangzhou, Jiangsu 225009, China; Department of Horticulture, School of Horticulture and Landscape Architecture, Yangzhou University, Yangzhou, Jiangsu 225009, China; Jiangsu Key Laboratory for Horticultural Crop Genetic Improvement, Institute ofVegetable Crops, Jiangsu Academy of Agricultural Sciences, Nanjing, Jiangsu 210014, China; Department of Horticulture, School of Horticulture and Landscape Architecture, Yangzhou University, Yangzhou, Jiangsu 225009, China; Jiangsu Key Laboratory for Horticultural Crop Genetic Improvement, Institute ofVegetable Crops, Jiangsu Academy of Agricultural Sciences, Nanjing, Jiangsu 210014, China

## Abstract

Abiotic stressors like waterlogging are detrimental to cucumber development and growth. However, comprehension of the highly complex molecular mechanism underlying waterlogging can provide an opportunity to enhance cucumber tolerance under waterlogging stress. We examined the hypocotyl and stage-specific transcriptomes of the waterlogging-tolerant YZ026A and the waterlogging-sensitive YZ106A, which had different adventitious rooting ability under waterlogging. YZ026A performed better under waterlogging stress by altering its antioxidative machinery and demonstrated a greater superoxide ion (O ^2−^) scavenging ability. KEGG pathway enrichment analysis showed that a high number of differentially expressed genes (DEGs) were enriched in phenylpropanoid biosynthesis. By pairwise comparison and weighted gene co-expression network analysis analysis, 2616 DEGs were obtained which were categorized into 11 gene co-expression modules. Amongst the 11 modules, black was identified as the common module and yielded a novel key regulatory gene, *CsPrx73*. Transgenic cucumber plants overexpressing *CsPrx73* enhance adventitious root (AR) formation under waterlogging conditions and increase reactive oxygen species (ROS) scavenging. Silencing of *CsPrx73* expression by virus-induced gene silencing adversely affects AR formation under the waterlogging condition. Our results also indicated that *CsERF7-3*, a waterlogging-responsive ERF transcription factor, can directly bind to the ATCTA-box motif in the *CsPrx73* promoter to initiate its expression. Overexpression of *CsERF7-3* enhanced *CsPrx73* expression and AR formation. On the contrary, *CsERF7-3*-silenced plants decreased *CsPrx73* expression and rooting ability. In conclusion , our study demonstrates a novel *CsERF7-3*–*CsPrx73* module that allows cucumbers to adapt more efficiently to waterlogging stress by promoting AR production and ROS scavenging.

## Introduction

Hypoxia and ethylene production from waterlogging impede root respiration and stunt plant growth [1, 2]. In response to waterlogging stress, plants employ various mechanisms. These include morphological changes, reactive oxygen species (ROS) scavenging, and hypoxia-responsive gene regulation. Thus, waterlogging disrupts the dynamic balance of ROS in plant cells [3], which causes an outburst and in the process increased peroxidation, membrane lipid peroxidation, and cell dysfunction. Plants under waterlogging stress rely on the antioxidant enzyme system and other active antioxidant substances to maintain the dynamic balance of ROS [4–6]. Globally, flooding affects ~27% of arable land each year, with yearly flood damage expenditures topping out at US$19 billion over the past 50 years. The climate change-induced intensification of the global water cycle seems to be leading to a larger incidence of waterlogging, exerting pressure on water usage efficiency and productivity of plants [2]. So, in order to sustain productivity, it is crucial to explore the mechanisms of plant waterlogging tolerance.

Adventitious roots (ARs) are rooting that sprout in plants from non-root tissues. They can be formed during normal development or in reaction to stressful circumstances including waterlogging, nutrient deficiency, or wounding [7]. Plants usually survive waterlogging stress by developing ARs, forming aerenchyma, metabolizing energy, and signaling phytohormones [8, 9]. Cucumber genotypes respond to waterlogging stress differently in terms of AR formation; these ARs improve gas exchange, nutrient uptake, and replace primary roots that die under waterlogging conditions [10, 11]. ARs have been observed across several plants, including sunflower [12], sugarcane [13], rice [14], maize [15], tomato [16], *Solanum dulcamara* [17], and cucumber [10], as a crucial component of waterlogging tolerance. The mechanism of waterlogging-triggered ARs often involves the balance of synthesis and transport of various endogenous phytohormones and regulates the response to waterlogging via a complex signaling network [8]. Several hormones positively regulate the formation of waterlogging-triggered ARs, including ethylene and auxin. Qi *et al*. [18] found that treating cucumber seedlings with 1-methylcyclopropene (1-MCP, an ethylene receptor inhibitor) inhibited the formation of ARs, whereas exogenous ACC (1-aminocyclopropane-1-carboxylic acid) promoted the formation of ARs under waterlogging. In rice, the auxin signaling pathway is involved in the coordinated process of AR occurrence and epidermal cell death [19]. In tomato, auxin accumulation indirectly triggers ethylene synthesis in the stem, which further induces the growth of ARs [20]. In contrast to ethylene and auxin, abscisic acid negatively regulates AR formation under waterlogging [17]. However, the regulatory effect of jasmonic acid on waterlogging stress appears inconsistent among different plant species [21, 22].

All living organisms contain peroxidases (Prxs), which catalyze the oxidation of certain substrates by hydrogen peroxide (H_2_O_2_), decreasing H_2_O_2_ concentrations inside plant tissues [23]. The *Arabidopsis*, rice, *Populus*, and Chinese pear genomes contain 73, 138, 93, and 94 Class III *PRX*s, respectively [24–27]. Class III Prx (EC 1.11.1.7) is a member of the plant-specific large multigene families belonging to the heme oxidoreductase subfamily [28, 29]. As a secretory peroxidase of higher plants, Class III Prx is fundamentally involved in the entire life cycle of plants, from seed germination to senescence [30, 31]. Class III Prx regulates plenty of physiological and developmental processes, including the removal of H_2_O_2_ [32], ROS, and reactive nitrogen species metabolism [33], auxin catabolism [34], cell metabolism, and synthesis of lignin and other cell wall components [35, 36]. In addition to its physiological function, Class III peroxidase operates as an antioxidant, which is crucial for coping with a variety of biotic and abiotic stresses. These stresses include adaptation to cold, drought, and salt stress [37, 38], tolerance of aluminum, zinc, cadmium, copper, and arsenic [39–42], and resistance to pathogenic diseases [43, 44].

The highly nutritious crop cucumber (*Cucumis sativus* L.), grown for its juicy fruit, is particularly vulnerable to waterlogging because of its shallow root structure and high demand for oxygen [45]. Monsoon rains in spring cause waterlogging conditions in the Yangtze River Delta region and the south of China, impairing cucumber crop production in open fields and greenhouses. Therefore, it is vital to investigate the molecular mechanism behind cucumber tolerance of waterlogging in order to mitigate the detrimental impact of waterlogging on cucumbers [10]. Unfortunately, there is a lack of research to fully comprehend the cucumber hypocotyl’s extensive transcriptional response to various waterlogging stress periods. Here we identified the European greenhouse-type cucumber inbred line YZ026A as having strong waterlogging tolerance, whereas the Northern China type cucumber inbred line YZ106A has weak waterlogging tolerance. Line YZ026A generated many ARs whereas the YZ106A generated almost no ARs. We obtained high-resolution hypocotyl and stage-specific transcriptome profiles from four stress stages using hypocotyl samples from YZ026A and YZ106A. Our research will provide useful genetic resources for the discovery of genes crucial for the development of waterlogging-triggered ARs and will provide high-quality genetic resources for subsequent breeding initiatives.

## Results

### Phenotypes of YZ026A and YZ106A upon waterlogging treatment

**Figure 1:**
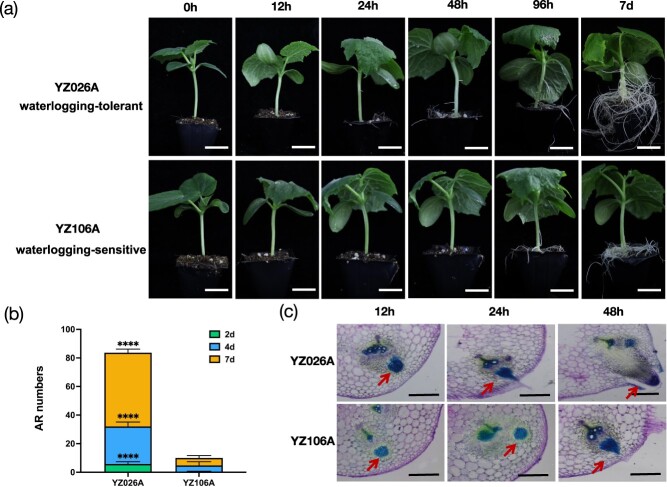
Comparison of morphology and anatomy between waterlogging-tolerant YZ026A and waterlogging-sensitive YZ106A. **a** Formation of ARs on hypocotyls at 0, 12, 24, 48, and 96 h and 7 days of waterlogging treatment. Scale bar = 2 cm. **b** number of ARs formed on hypocotyls of YZ026A and YZ106A at 2, 4, and 7 days of waterlogging. **c** Cross-sections of YZ026A and YZ106A hypocotyls at 12, 24, and 48 h of waterlogging. The arrow indicates AR primordia. Each value represents the mean ± standard deviation. ^****^P < 0.0001.

To determine the difference in AR primordia between waterlogging-tolerant YZ026A and waterlogging-sensitive YZ106A [46], 15-day-old seedlings were exposed to waterlogging. Variations in AR formation of YZ026A and YZ106A at five different stages (12, 24, 48, 96, and 168 h) were observed (Fig. 1a). YZ026A hypocotyls produced a considerable number of ARs, but YZ106A hypocotyls generated no ARs (Fig. 1b). Histological analysis revealed that the AR primordia first appeared in the vascular cambium region of YZ026A hypocotyls and broke through the hypocotyl epidermis after 48 h of waterlogging treatment; however, this was not apparent in YZ106A even after 48 h of waterlogging treatment (Fig. 1c).

### RNA-seq analysis of hypocotyls upon waterlogging treatment

**Figure 2:**
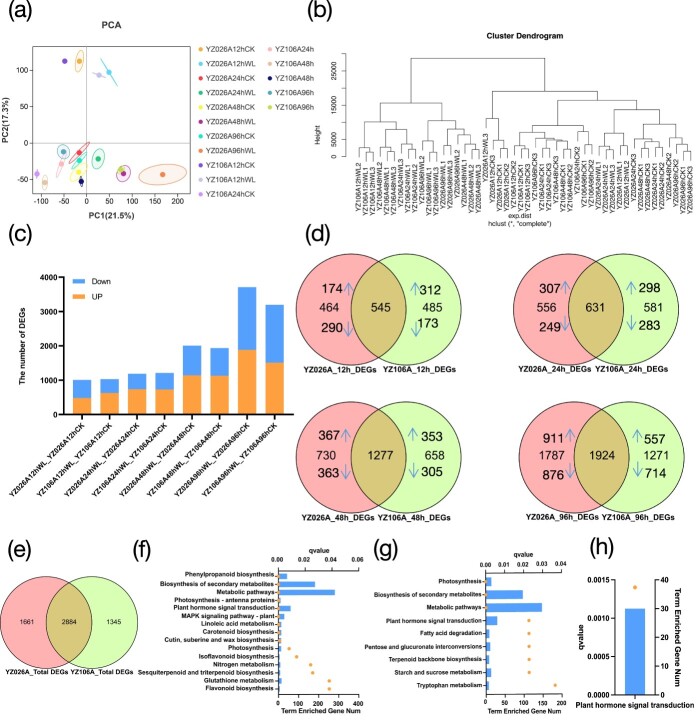
Overview of RNA-seq data. **a** PCA of genes that were found in all samples. **b** Graph representing the samples’ hierarchical grouping based on the normalized FPKM values for all detected genes. **c** Numbers of up- and downregulated DEGs in four waterlogging stress stages in YZ026A and YZ106A compared with the relative non-waterlogged controls. **d** Venn diagrams of DEGs induced by waterlogging in YZ026A and YZ106A compared with control treatment in the four waterlogging stress stages. A down arrow indicates a downregulated DEG, whereas an up arrow indicates an upregulated one. **e** Venn diagram of total DEGs induced by waterlogging in YZ026A and YZ106A compared with control treatment. **f** KEGG pathway analysis of 2884 DEGs in (**e**). **g** KEGG pathway analysis of 1661 DEGs in (**e**). **h** KEGG pathway analysis of 1345 DEGs in (**e**). The *x*-axis represents the *q*-value and enriched gene numbers. The yellow dot represents the *q* value and the blue box represents enriched gene number.

RNA-seq analysis was performed using hypocotyl samples from YZ026A and YZ106A harvested at 12, 24, 48, and 96 h of waterlogging treatment. A total of 2184.7 Mb clean reads were obtained with an average of 45.5 Mb clean reads per sample (Supplementary Data Table S1). However, match ratios ranged from 96.5 to 97.67%, and 38.9 to 46.6 million reads mapped in correct pairs to the cucumber genome (9930_v3). The RNA-seq data were subjected to principal component analysis (PCA), which revealed that these points were well separated. The first and second components explain 21.5 and 17.3% of the variability in the dataset, respectively (Fig. 2a). By separating the three biological replicates by time point, treatment, and genotype, hierarchical clustering analysis demonstrated that they were clustered together, further supporting the dependability of our RNA-seq results (Fig. 2b). The clustering was more pronounced amongst samples with waterlogging durations of 12, 24, 48, and 96 h, suggesting that waterlogging duration may be the primary factor affecting gene expression. At 12 and 24 h, there was minimal difference between waterlogging treatments and the non-waterlogged control, and the distances between YZ026A and YZ106A were also rather close. The separation between the YZ026A and its control, however, was greater at 48 and 96 h than it was between the YZ106A and its control. To assess the reliability of the RNA-seq results, six genes were randomly chosen and subjected to the real-time quantitative polymerase chain reaction (qRT–PCR) Both qRT–PCR and RNA-seq analysis showed consistent gene expression levels (*R*^2^ = 0.7353), confirming the reliability of the transcriptome analysis (RNA-seq) data (Supplementary Data Fig. S1).

### Characteristics of differentially expressed genes in YZ026A and YZ106A

Using a fold change of ≥2 and *P* ≤ 0.05 as cut-off, a total of 4545 and 4229 no-overlap waterlogging-responsive differentially expressed genes (DEGs) were identified in YZ026A and YZ106A, respectively. In detail, 1009 (482 upregulated and 527 downregulated), 1187 (735 upregulated and 452 downregulated), 2007 (1142 upregulated and 865 downregulated), and 3711 (1884 upregulated and 1827 downregulated) DEGs were identified in YZ026A at 12, 24, 48, and 96 h of waterlogging, respectively (Fig. 2c). In contrast, 1030 (628 upregulated and 402 downregulated), 1212 (731 upregulated and 481 downregulated), 1935 (1131 upregulated and 804 downregulated), and 3195 (1511 upregulated and 1684 downregulated) DEGs were identified in YZ106A at 12, 24, 48, and 96 h of waterlogging, respectively (Fig. 2c). The findings demonstrated that both lines had an increase in the number of DEGs during the course of prolonged treatment, with YZ026A showing a greater DEG response to waterlogging than YZ106A, particularly after 96 h. All the DEGs found for each pairwise comparison are listed in Supplementary Data Table S2.

At 12, 24, 48, and 96 h of waterlogging treatment, the two lines shared 545, 631, 1277, and 1924 DEGs, respectively (Fig. 2d). According to our current results, waterlogging stress causes a transcriptional reprogramming of numerous common genes. In both cultivars, waterlogging stress co-regulated 2884 of these DEGs (Fig. 2e). The 2884 DEGs were analyzed using KEGG and found to be significantly enriched in 15 pathways. These pathways include phenylpropanoid biosynthesis (ko00940), biosynthesis of secondary metabolites (ko01110), metabolic pathways (ko01100), photosynthesis-antenna proteins (ko00196), plant hormone signal transduction (ko04075), MAPK signaling pathway (ko04016), linoleic acid metabolism (ko00591), and carotenoid biosynthesis (ko00906) (Fig. 2f). For the 1661 DEGs that were specifically expressed in YZ026A, nine pathways were significantly enriched, including photosynthesis (ko00195), biosynthesis of secondary metabolites (ko01110), metabolic pathways (ko01100), plant hormone signal transduction (ko04075), fatty acid degradation (ko00071), pentose and glucuronate interconversions (ko00040), terpenoid backbone biosynthesis (ko00900), starch and sucrose metabolism (ko00500), and tryptophan metabolism (ko00380) (Fig. 2g). For the 1345 DEGs that were specifically expressed in YZ106A, only one pathway was significantly enriched, which was plant hormone signal transduction (ko04075) (Fig. 2h).

**Figure 3:**
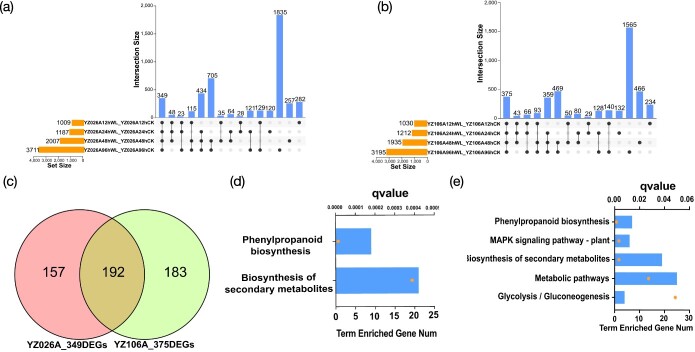
The ‘core’ set of DEGs was identified and analyzed through the pathway analysis process. **a** Upset Venn diagram of DEGs at the four time points in YZ026A. **b** Upset Venn diagram of DEGs at the four time points in YZ106A. **c** Venn diagram of 349 DEGs in (**a**) and 375 DEGs in (**b**). **d** KEGG pathway analysis of 157 DEGs specific to YZ026A. The *y*-axis represents the *q*-value and enriched gene numbers. **e** KEGG pathway analysis of 192 DEGs in both YZ026A and YZ106A. The *x*-axis represents the *q*-value and enriched gene numbers. The yellow dot represents the *q* value and the blue box represents enriched gene number.

We analyzed the DEGs in YZ026A and YZ106A using a Venn diagram to find shared or individual genes implicated in waterlogging stress across all four time points (Fig. 3a and b). Most DEGs were temporarily differentially induced by waterlogging stress at specific time points. For example, 1835 of the 4545 DEGs in YZ026A and 1565 of the 4229 DEGs in YZ106A showed differential expression only at 96 h, while a relatively small number of DEGs (349 and 375) overlapped at all four time points in YZ026A and YZ106A, respectively (Fig. 3a and b). We performed a Venn diagram analysis on 349 DEGs in YZ026A and 375 DEGs in YZ106A (Fig. 3c) and found that 157 DEGs were only differentially expressed in the waterlogging-tolerant YZ026A. KEGG enrichment analysis showed that two pathways, phenylpropanoid biosynthesis (ko00940) and biosynthesis secondary metabolites (ko01110) were significantly enriched (Fig. 3d). Consistently, the Gene Ontology (GO) enrichment analysis showed that heme binding (GO:0020037) and hydrogen peroxide catabolic process (GO:0042744) were the most significantly enriched GO terms (Supplementary Data Table S3). At each of the four time points, 192 DEGs were found to overlap (Fig. 3e), suggesting that these genes were differentially expressed in both cultivars over time. Interestingly, phenylpropanoid biosynthesis (ko00940) was also the most significantly enriched pathway. One hundred and eighty-three DEGs were specifically differentially expressed in YZ106A, and, surprisingly, none of the KEGG enrichment pathways were significant (*q* > 0.05) (Supplementary Data Table S4). Together, our RNA-seq data showed that YZ026A exhibits more abundant and significant transcriptional changes in a hypoxic environment than YZ106A, with high numbers of genes associated with phenylpropanoid biosynthesis and biosynthesis of secondary metabolites.

### Weighted gene co-expression network analysis revealed waterlogging-responsive peroxide pathways

**Figure 4:**
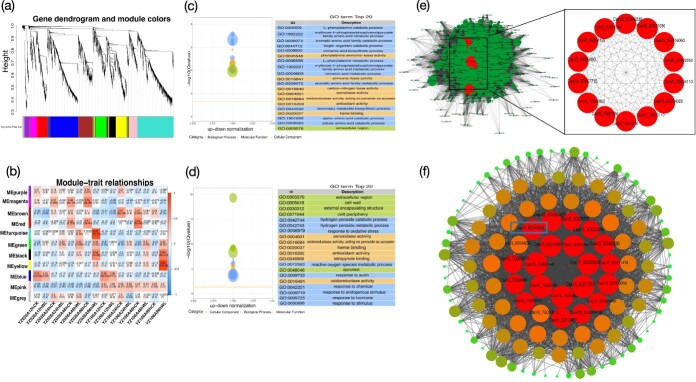
Evaluation of weighted gene co-expression networks and display of important modules’ co-expression networks. **a** Co-expression modules are arranged in a hierarchical grouping tree. Every leaf on the tree represents one gene. A total of 2621 genes yielded 11 co-expression modules marked by different colors. **b** Each line represents one module. Each column represents one type of sample. The color of each cell indicates the correlation coefficient and *P* value between the module and the specific sample. The table is color-coded according to the color scale on the right (from blue for − 1 to red for 1). **c** GO functional enrichment categories of significantly correlated black modules. Only the top 20 terms are displayed. **d** GO functional enrichment categories of significantly correlated turquoise modules. Only the top 20 terms are displayed. **e** Co-expression network of the turquoise module. There is a gene for every node, and the edges that link genes show the correlations in their co-expression. Among the 639 genes shown in the network, key hub genes are indicated with large circles and red color. **f** Co-expression network of black module, with a total of 162 genes. The genes in the inner two circles represent the hub gene identified based on WGCNA analysis.

Using weighted gene co-expression network analysis (WGCNA), we looked into the regulatory genes of the waterlogging response and found groupings of co-expression genes [47]. The analysis grouped the 2616 genes into 11 distinct modules (marked with distinct colors), which comprised 34 (gray) to 639 (turquoise) genes in each module (Fig. 4a, details in Supplementary Data Tables S5 and S6). In the black module, the expressions of 162 clustered genes were found to increase upon waterlogging treatment in both of the two lines (Fig. 4b, Supplementary Data Table S6). An abundance of GO terms pertaining to the catabolic processes of l-phenylalanine catabolic process (GO:0006559), erythrose 4-phosphate/phosphoenolpyruvate family amino acid catabolic process (GO:1902222), aromatic amino acid family catabolic process (GO:0009074), single-organism catabolic process (GO:0044712), and cinnamic acid biosynthetic process (GO:0009800) were significantly enriched (Fig. 4c). For the turquoise module, the clustered genes were only differently expressed in YZ026A (Fig. 4b). GO enrichment analysis showed that genes in the turquoise module were mainly related to peroxide-related metabolic pathways, which included 18 genes in H_2_O_2_ catabolic process (GO:0042744), 19 genes in H_2_O_2_ metabolic process (GO:0042743), and 32 genes in response to oxidative stress (GO:0006979) (Fig. 4d, Supplementary Data Table S7). The findings strongly imply that YZ026A might alter H_2_O_2_ metabolism and antioxidant pathways to adapt to waterlogging stress.

The turquoise and black modules’ key regulatory genes were further identified by creating and visualizing a gene network with Cytoscape [48]. In this network, every node stands for a gene, and the edges that link them show the correlations in their co-expression. The most connected hub genes in this network are likely to be the key regulatory genes. The core genes of the modules were screened based on high weight and high degree values. Turquoise and black modules were screened with 14 and 22 core genes, respectively. According to the gene interaction network of the turquoise module, the highly connected genes mainly included *CsaV3_1G033110* (WRKY), *CsaV3_2G034110* (peroxidase), *CsaV3_1G0107*00 (glyoxalase), *CsaV3_3G021030* (glutamine synthetase), and *CsaV3_3G042330* (sugar efflux transporter for intercellular exchange) (Fig. 4e, Supplementary Data Table S8). The hub genes in the black module mainly include *CsaV3_6G043930* and *CsaV3_7G006370* (peroxidase), *CsaV3_7G030110* (WRKY), *CsaV3_3G011470* (glutathione *S*-transferase), *CsaV3_3G040830* (calcineurin-like phosphoesterase), *CsaV3_2G029490* (gibberellin regulated protein), and *CsaV3_3G023620* (ABC-2 type transporter) (Fig. 4f, Supplementary Data Table S8).

Based on the results of transcriptomic analysis, we found the peroxide-related metabolic pathway is involved in waterlogging stress. We thus evaluated the antioxidant enzyme activities in YZ026A and YZ106A upon waterlogging treatment. A significant increase in peroxidase (POD) activity was observed in YZ026A hypocotyls at 96 h of waterlogging treatment. On the contrary, a significant decrease in POD activities was observed in waterlogged YZ106A at 24 and 48 h (Supplementary Data Fig. S2a). The fluctuations in catalase (CAT) activities were essentially the same as those in POD, indicating that the waterlogging-tolerantYZ026A maintained a dynamic equilibrium between the levels of POD and CAT *in vivo* when subjected to waterlogging stress, thereby decreasing damage to the plants (Supplementary Data Fig. S2b). Superoxide dismutase (SOD) activities in YZ026A increased gradually with waterlogging treatment duration, peaking at 96 h (Supplementary Data Fig. S2c), but SOD activities in YZ106A were considerably elevated only at 96 h (Supplementary Data Fig. S2c). Additionally, there was no significant change in H_2_O_2_ content in YZ026A until 96 h of treatment, whereas it was accumulated significantly in YZ106A from 24 h of treatment (Supplementary Data Fig. S2d). These results demonstrate that YZ026A had decreased oxidative damage.

### Lignin biosynthesis plays an important role in waterlogging tolerance

**Figure 5:**
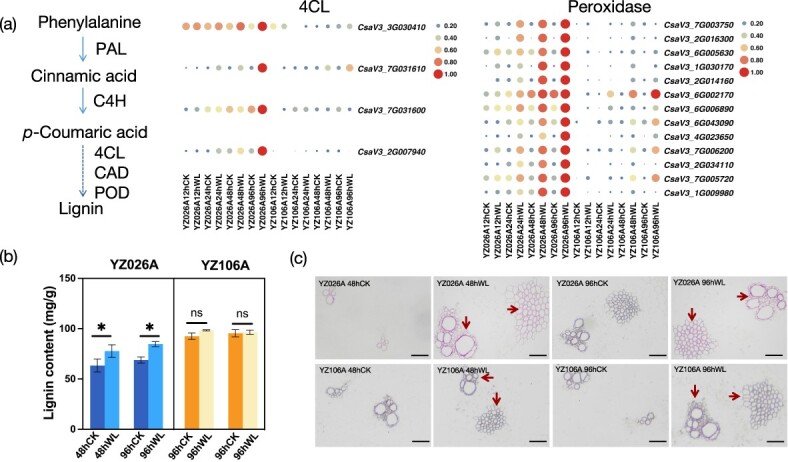
The lignin biosynthesis pathway is involved in waterlogging treatment. **a** Heat map displaying the DEGs that contribute to lignin biosynthesis. **b** Effects of waterlogging stress on the lignin content in hypocotyl of cucumber. **c** Phloroglucinol staining of hypocotyl cross section in paraffin. Scale bar =100 μm. Data represent mean ± standard deviation (three biological replicates, nine plants for per replicate). The mean ± standard deviation of three biological replicates is represented by each value. ^*^P < 0.05; ns, non-significant mean difference.

The phenylpropanoid biosynthesis pathway was significantly enriched in the turquoise module (Supplementary Data Fig. S3). A total of 19 genes were found to be associated with lignin biosynthesis, including one gene encoding cinnamate-4-hydroxylase (C4H) (*CsaV3_1G026620*), four genes encoding *trans*-cinnamate 4-monooxygenase 4-coumarate–CoA ligase (4CL) (*CsaV3_3G030410*, *CsaV3_7G031610*, *CsaV3_7G031600*, *CsaV3_2G007940*), one gene encoding cinnamyl alcohol dehydrogenase (CAD) (*CsaV3_2G018020*), and 13 genes encoding peroxidase (Prx) (*CsaV3_7G003750*, *CsaV3_2G016300*, *CsaV3_6G005630*, *CsaV3_1G030170*, *CsaV3_2G014160*, *CsaV3_6G002170*, *CsaV3_6G006890*, *CsaV3_6G043090*, *CsaV3_4G023650*, *CsaV3_7G006200*, *CsaV3_2G034110*, *CsaV3_7G005720*, *CsaV3_1G009980*) (Supplementary Data Table S9). Furthermore, as seen in Fig. 5a, waterlogging treatment greatly increased the expression of the 19 genes identified. Thus, the lignin contents were determined, and the findings revealed that lignin contents considerably increased in YZ026A hypocotyls after waterlogging treatment, whereas they remained unaffected in YZ106A hypocotyls at 48 and 96 h after waterlogging treatment (Fig. 5b). Histochemical staining was used to further observe the lignin deposition, and the results showed that YZ026A’s vascular cambium showed a darker color after waterlogging, which is consistent with increased lignin content, while YZ106A exhibited no apparent shift after waterlogging (Fig. 5c).

### Transgenic *CsPrx73* exhibits better rooting ability

**Figure 6:**
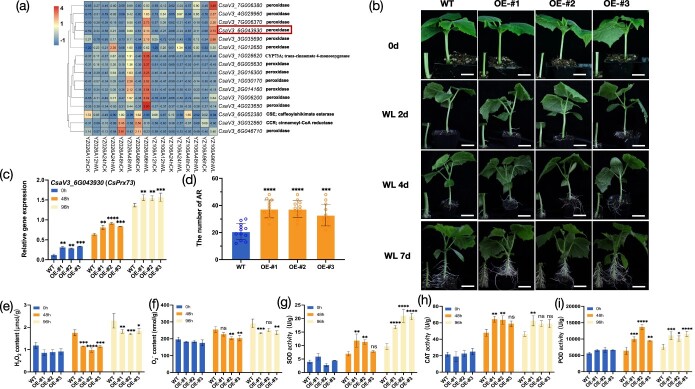
Expression of *CsPrx73*. **a** Heat map showing the relative FPKM of genes in the phenylpropanoid biosynthesis pathway from 192 overlapped DEGs at all four time points. **b** Phenotype of three transgenic *CsPrx73* (OE-#1, OE-#2, and OE-#3) lines and WT plants at 0, 2, 4, and 7 days of waterlogging treatment. Scale bar = 3 cm. **c***CsPrx73* relative expression level in transgenic *CsPrx73* lines and WT plants. **d** Number of ARs in transgenic *CsPrx73* lines and WT plants on the 7th day of waterlogging stress. **e** H_2_O_2_ content. **f** O^2−^ content. **g**–**i** ROS scavenger activity in hypocotyls of WT and *CsPrx73*-OE plants. The mean ± standard deviation of three biological replicates is represented by each value. ^*^P < 0.05; ^**^P < 0.01; ^***^P < 0.001; ^****^P < 0.0001; ns, not significant.

Based on FPKM (fragments per kilobase of transcript per million mapped fragments) values, we analyzed the expression of the genes in the phenylpropanoid biosynthesis pathway after waterlogging treatment. The phenylpropanoid synthesis pathway included 13 genes encoding Prx (Fig. 6a). Among them, *CsV3_6G043930* was selected for further analysis; it had the highest FPKM under waterlogging stress and was a hub gene in the black module (Fig. 4f). *CsaV3_6G043930* was the cucumber homolog of *Arabidopsis* Class III peroxidase 73, designated as *CsPrx73*.

To investigate the relationship between *CsPrx73* expression and waterlogging stress, we overexpressed *CsPrx73* in the cucumber inbred line 9930. A total of three independent *T*_2_ transgenic lines, OE-#1, OE-#2, and OE-#3, 3, (Fig. 6b) were obtained (Supplementary Data Fig. S4) and the expressions of *CsaV3_6G043930* in the three transgenic lines were all significantly increased when compared with wild-type (WT) (Fig. 6c). Plants with two true leaves were given the waterlogging treatment and 7 days later the number of ARs was recorded. The ARs emerging from the hypocotyl of transgenic lines OE-#1 (37.42 ± 6.60), OE-#2 (37.33 ± 6.18), and OE-#3 (32.92 ± 8.03) were observed to be substantially greater than those of WT plants (20.75 ± 5.99) (Fig. 6b and d). Furthermore, after being exposed to waterlogging, transgenic *CsPrx73* plants had considerably lower O_2_^−^ and H_2_O_2_ levels (Fig. 6e and f) and higher SOD, POD, and CAT activities than WT plants (Fig. 6g–i). Finally, our findings show that *CsPrx73* promotes waterlogging tolerance by regulating the antioxidative stress mechanism and ROS scavenging abilities. It should be noted that no significant change in lignin contents was observed in transgenic *CsPrx73* plants, despite lignin biosynthesis-related *Cs4CL* genes being significantly upregulated (Supplementary Data Fig. S5).

**Figure 7:**
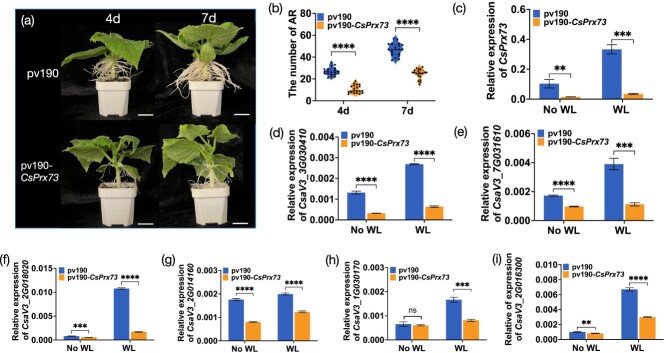
Phenotype of 3-week-old plants infected with empty pV190 vector or pV190-*CsPrx73* that were waterlogged for 4 or 7 days and the expression of lignin biosynthesis-related genes in pv190-*CsPrx73* plants and empty pv190 plants. **a** Three-week-old empty plants infected with pV190 vector or pV190-*CsPrx73* that were waterlogged for 4 or 7 days. Scale bar = 2 cm. **b** Number of ARs in plants infected with empty pV190 vector or pV190-*CsPrx73* after waterlogging for 4 or 7 days. **c** qRT–PCR analysis of *CsPrx73* in the hypocotyls of Superina plants infiltrated with *A. tumefaciens* carrying the pV190-*CsPrx73* or empty pV190 vector without waterlogging (No WL) and at 48 h after waterlogging treatment. **d**–**i** Expression of lignin biosynthesis-related genes in plants infected with empty pV190 vector or pV190-*CsPrx73*. *CsaV3_3G030410* and *CsaV3_7G031610* encode 4CL; *CsaV3_2G018020* encodes CAD; *CsaV3_2G014160*, *CsaV3_1G030170*, and *CsaV3_2G016300* encode peroxidase (Prx). Each value represents the mean ± standard deviation of three biological replicates. ^**^P < 0.01; ^***^P < 0.001; ^****^P < 0.0001; ns, non-significant mean difference.

To further confirm the role of *CsPrx73* in rooting ability, *CsPrx73* was silenced using virus-induced gene silencing (VIGS). At 4 and 7 days after waterlogging stress, the number of ARs on pV190-*CsPrx73* plants was lower. with a substantial decrease in *CsPrx73* compared with empty vector pV190 plants (Fig. 7a and b). The expression of *CsPrx73* was significantly suppressed in pV190-*CsPrx73* (Fig. 7c). Moreover, we observed substantial downregulation of the lignin biosynthesis-related genes (*CsaV3_3G030410*, *CsaV3_7G031610*, *CsaV3_2G018020*, *CsaV3_2G014160*, and *CsaV3_2G016300*) in the pV190-*CsPrx73* plants 48 h after waterlogging stress and in the absence of waterlogging stress (Fig. 7d–i).

### *CsERF7-3* binds to and activates the *CsPrx73* promoter

**Figure 8:**
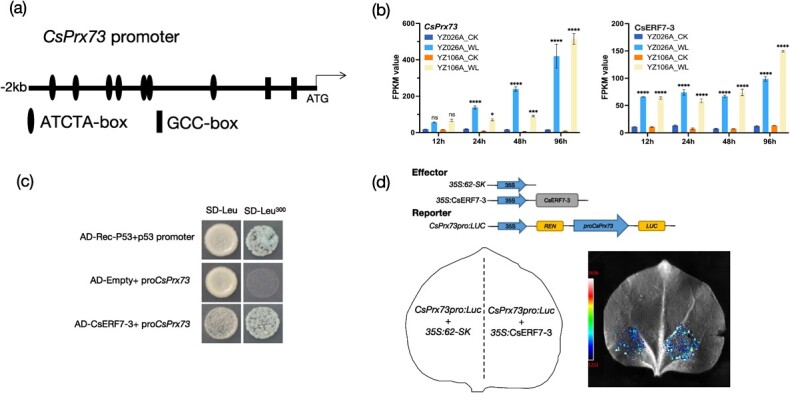
*CsERF7-3* binds to the *CsPrx73* promoter and activates *CsPrx73* expression. **a** Graphic representation of cucumber *CsPrx73* promoter with a 2-kb region upstream of the coding sequence. Seven putative *CsERF7-3* binding sites (ATCTA-box, ATCTA/TAGAT) are marked by ellipses and two GCC-boxes (GCCGCC/GGCGGC) are marked by rectangles . **b***CsPrx73* and *CsERF7-3* displayed an upregulated expression pattern in YZ026A under the waterlogged condition. **c***CsERF7-3* binding to the *CsPrx73* promoter in the yeast one-hybrid assay. AD-Empty, pGADT7 vector; AD-CsERF7-3, pGADT7-*CsERF7-3*. pGAD-Rec2-53 is a positive control vector that encodes murine p53 as a fusion with the GAL4 AD. **d** Diagram depicting effector and reporter components utilized in the dual-luciferase experiment. In *Nicotiana benthamiana* cells, the CsERF7-3 protein stimulates transcription of the LUC (luciferase) reporter gene, which is driven by the 2-kb p*CsPrx73* promoter. The mean ± standard deviation of three biological replicates is represented by each value. ^*^P < 0.05; ^***^P < 0.001; ^****^P < 0.0001; ns, non-significant mean difference.

To investigate the upstream transcription factor (TF) governing *CsPrx73* transcription, we searched the *CsPrx73* promoter for particular DNA sequences that could be recognized by TF(s). We found seven ethylene-responsive *cis-*acting elements (core ATCTA or TAGAT in the opposite strand) and two GCC-box elements (GCCGCC or GGCGGC in the opposite strand) in the 2 kb upstream promoter region of *CsPrx73*, which were putative *cis*-acting elements for ethylene-responsive factor (ERF; Fig. 8a, Supplementary Data Fig. S6) [49, 50]. Interestingly, we found an ERF, *CsEFR7-3* (*CsaV3_4G000990*), in the same module (MEblack) with *CsPrx73* (Fig. 8b).

To confirm the interaction between *CsEFR7-3* and the *CsPrx73* promoter, we employed a yeast one-hybrid (Y1H) assay. Bait-containing yeast cells grew normally on selective a medium containing 300 ng/ml aureobasidin A (AbA) when co-transformed with *CsPrx73* promoter (bait) or positive control but were completely inhibited when co-transformed with the empty vector (Fig. 8c). To explore whether *CsEFR7-3* regulates *CsPrx73* promoter activity *in planta*, we conducted a dual-luciferase (LUC) assay. Compared with empty vector control, the co-expression of 35S::*CsERF7-3* and p*CsPrx73*::0800-LUC resulted in a significant increase in LUC activity (Fig. 8d). These results indicate that *CsERF7-3* positively regulates C*sPrx73* activity by binding to its promoters.

### *CsERF7-3* is required for waterlogging-triggered adventitious root formation

**Figure 9:**
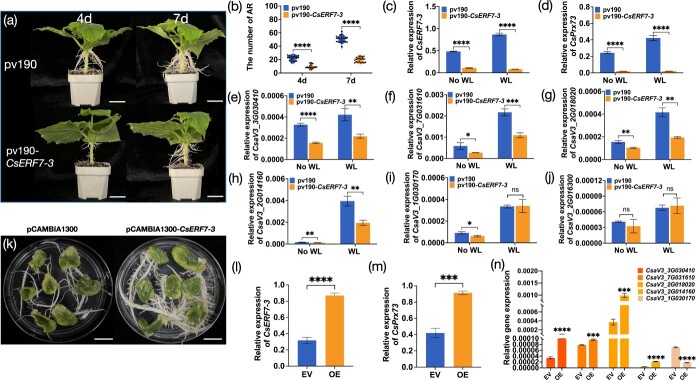
*CsERF7-3* positively regulates adventitious root formation. **a** Representative phenotypes of Superina plants injected with the VIGS-CK (empty pV190 vector) or VIGS-*CsERF7-3* vector. Plants were phenotyped at 4 and 7 days (d) after waterlogging treatment. Scale bar = 2 cm. **b** Number of ARs in plants infected with empty pV190 vector or pV190-*CsERF7-3* after 4 and 7 days of waterlogging. **c**–**j** qRT–PCR analysis of *CsERF7-3* (**c**), *CsPrx73* (**d**), and lignin biosynthesis-related genes including *CsaV3_3G030410* (encoding 4-coumarate-CoA ligase, **e**), *CsaV3_7G031610* (encoding 4-coumarate-CoA ligase, **f**), *CsaV3_2G018020* (encoding cinnamyl alcohol dehydrogenase, **g**), *CsaV3_2G014160* (encoding peroxidase, **h**), *CsaV3_1G030170* (encoding peroxidase, **i**), and *CsaV3_2G016300* (encoding peroxidase, **j**) in the hypocotyls of Superina plants infiltrated with pV190-*CsERF7-3* or empty pV190 vector without waterlogging (No WL) or with 48 h of waterlogging treatment. (**k**) Phenotype of transgenic cucumber hairy roots expressing pCAMBIA1300 empty vector or pCAMBIA1300-*CsERF7-3* vector. Scale bar = 1.5 cm. **l**–**n** qRT–PCR analysis of *CsERF7–3* (**l**), *CsPrx73* (**m**), and lignin biosynthesis-related genes (**n**) in transgenic cucumber hairy roots expressing pCAMBIA1300 empty vector (EV) or pCAMBIA1300-*CsERF7-3* vector (OE). The mean ± standard deviation of three biological replicates is represented by each value. ^*^P < 0.05; ^**^P < 0.01; ^***^P < 0.001; ^****^P < 0.0001; ns, non-significant mean difference.

The VIGS assay was used to investigate the possible involvement of *CsERF7-3* in AR formation under waterlogging treatment. At 4 and 7 days after waterlogging stress, the pV190-*CsERF7-3*-infiltrated plants showed a notably reduced amount of AR compared with plants infected with pV190 control (Fig. 9a and b). The relative expression levels of *CsERF7-3* and *CsPrx73* were significantly downregulated in *CsERF7-3*-silenced plants, as confirmed by qRT–PCR (Fig. 9c and d). Furthermore, we found that the lignin biosynthesis-related genes (*CsaV3_3G030410*, *CsaV3_7G031610*, *CsaV3_2G018020*, and *CsaV3_2G014160*) were also downregulated in *CsERF7-3*-silenced plants (Fig. 9e–j).

To further confirm the function of *CsERF7-3*, we generated cucumber hairy roots overexpressing *CsERF7-3* using the *Agrobacterium rhizogenes* strain K599. Compared with the empty vector (pCAMBIA1300), a stronger increase in adventitious rooting ability in *CsERF7-3*-overexpressing roots was observed (Fig. 9k). The upregulation of *CsERF7-3* and *CsPrx73* expressions in *CsERF7-3*-overexpressing roots was also confirmed by qRT–PCR analysis (Fig. 9l and m).These results imply that the *CsPrx73* and *CsERF7-3* module plays a beneficial regulatory role in AR formation and root growth in cucumber plants. In addition, we found that the lignin biosynthesis-related genes (*CsaV3_3G030410*, *CsaV3_7G031610*, *CsaV3_2G018020*, and *CsaV3_2G014160*) were also significantly increased in *CsERF7-3*-overexpressing roots (Fig. 9n).

## Discussion

Unpredictable precipitation is becoming more severe, posing new challenges to global agriculture in the form of sudden flooding and waterlogged soil [51, 52]. Cucumber growth and development are increasingly threatened by waterlogging; therefore, there is a pressing need for waterlogging-tolerant cucumber varieties [53]. Despite numerous studies on cucumber’s response to waterlogging stress [18, 54], our comprehension of its molecular regulatory network remains limited. Here, we opted for the waterlogging-tolerant YZ026A, which produced a large number of ARs, and the waterlogging-sensitive YZ106A, which produced essentially no ARs, to examine the transcriptional dynamics of these genes over an extended waterlogging treatment (Fig. 1a and b). AR development on the hypocotyl is an essential morphological adaptation to waterlogging stress that can replace the primary roots that die in O_2_-deficient scenarios [45].

In response to submersion, deepwater rice produces AR primordia at each newly formed node [14]. Here, the AR primordia of YZ026A and YZ106A arose at 24 h and 48 h under waterlogged conditions, respectively (Fig. 1c). We built a complete high-resolution temporal dynamic transcriptome utilizing the hypocotyl tissues from the two lines to identify its gene regulation network. The analysis of dynamic transcriptome data clearly showed the differences between YZ026A and YZ106A at different time points in response to waterlogging stress. Our comprehensive transcriptome data offer an abundance of data for identifying critical genes involved in the development of ARs during waterlogging stress and will significantly advance our comprehension of how cucumbers react to waterlogging stress.

Our RNA-seq data indicated that many DEGs are involved in the transcriptional reprogramming responding to waterlogging stress. Of these, 1661 DEGs specific to YZ026A were significantly enriched in nine pathways (Fig. 2e and g). However, 1345 DEGs specific to YZ106A were significantly enriched in only one pathway (Fig. 2e and h). This revealed that YZ026A exhibited more abundant and significant transcriptional changes upon waterlogging stress. A relatively small number of DEGs overlapped at all time points in YZ026A and YZ106A, 349 and 375, respectively (Fig. 3a and b), suggesting that the transcriptome in the cucumber response to waterlogging was changing dynamically. Among them, 157 DEGs specific to YZ026A were significantly enriched in the phenylpropanoid biosynthesis pathway and biosynthesis of secondary metabolites pathway (Fig. 3d), while 183 DEGs were specifically expressed only in YZ106A, but the KEGG enrichment pathway was not significant (*q* > 0.05) (Supplementary Data Table S4). Overall, YZ026A showed more abundant and significant transcriptional changes to the hypoxia environment than YZ106A, with a high number of genes associated with phenylpropanoid biosynthesis and biosynthesis of secondary metabolites. YZ026A was more tolerant of the waterlogging environment than YZ106A, and the 157 DEGs specific to YZ026A were tightly associated with cucumber waterlogging tolerance, contributing to the AR differences upon waterlogging treatment between YZ026A and YZ106A.

Phenylpropanoid plays an important role in various aspects of plant responses to biotic and abiotic stress [55, 56]. Lignin is a component of plant phenylpropanoids, together with flavonols, anthocyanins, and proanthocyanidins [57]. As the main component of the plant cell wall, increased lignin deposition in the outer cell layers during AR maturation may form a barrier for radial O_2_ loss in rice [14]. In our study, the expressions of 19 DEGs related to the phenylpropanoid biosynthetic metabolic pathway were significantly upregulated in YZ026A, and these genes were mainly involved in lignin biosynthesis (Fig. 5a). Simultaneously, the lignin content in hypocotyls of YZ026A was significantly increased, whereas no significant change was observed in YZ106A after 48 and 96 h of waterlogging stress (Fig. 5b). This indicated that cucumber could resist the damage of waterlogging stress by increasing lignin content.

Adverse environmental conditions, including waterlogging stress, can lead to the overaccumulation of ROS, such as free radicals (superoxide anion, O_2_^•−^; hydroperoxyl radical, HO_2_^•^; alkoxy radical, RO^•^; and hydroxyl radical, ^•^OH) and non-radical molecules (H_2_O_2_ and O_2_^•−^), which eventually lead to oxidative damage to DNA, RNA, proteins, and membranes [58, 59]. Plants scavenge excess ROS produced by oxidative stress directly or indirectly through an antioxidant defense mechanism that comprises both non-enzymatic and enzymatic antioxidants to reduce ROS degradation in plant cells [60]. POD is one of the most important enzymes in the plant defense system among enzymatic antioxidants. It works in conjunction with CAT and SOD to eliminate excess free radicals and shield organisms from oxidative stress, enhancing plant stress tolerance [61]. Waterlogging stress increased SOD, POD, and CAT activities in YZ026A, which is similar to the findings of Li *et al*. [62]. ROS levels in waterlogged YZ106A hypocotyls were significantly greater than in YZ026A (Supplementary Data Fig. S2). Higher ROS-scavenging enzyme activity and lower ROS accumulation may account for YZ026A’s waterlogging tolerance.

Prxs act as major ROS scavengers and can be divided into three subgroups: Class I (ascorbate), Class II (lignin), and Class III (secretory) [63, 64]. Members of Class III Prxs are involved in abiotic stress, according to numerous studies. The *GsPRX9* gene is overexpressed, which improves soybean salt tolerance and antioxidant response [65]. The survival rate of wheat overexpressing *TaPRX-2A* was higher than that of WT with augmented relative water content and stem length. Compared with WT, SOD, POD, and CAT activities increased, and ROS and malondialdehyde decreased in *TaPRX-2A-*overexpressing wheat [66]. In sweet potato, the B-box family TF *IbBBX24* activates the expression of *IbPRX17* by binding to its promoter and overexpression of *IbPRX17* leads to salt and drought stress tolerance [67]. Thus, plants have evolved an enzymatic defense system that allows them to maintain cellular ROS equilibrium while responding to and adapting to environmental stimuli. *CsPrx73* expression was found to be increased in YZ026A and YZ106A in our study. Furthermore, 13 peroxidase genes were selectively activated by waterlogging stress in YZ026A, showing that YZ026A has stronger antioxidant activity than YZ106A and the *CsPrx73* gene may play an essential role in improving the waterlogging tolerance of YZ026A (Fig. 6a). *CsPrx73* was most closely related to *AtPRX73*, which is required for ROS-mediated root hair growth [68]. In the present study, overexpression of *CsPrx73* improved waterlogging tolerance by promoting AR formation (Fig. 6b and c), increasing antioxidant enzyme activities and decreasing ROS levels relative to WT plants (Fig. 6e–i). These findings imply that *CsPrx73* regulates waterlogging tolerance positively by limiting ROS production.

Members of the ERF family regulate stress responses primarily by binding to specific promoter sequences of resistance-related genes (*cis*-acting GCC boxes) [62]. Despite the fact that several stress-associated members have been shown to bind to GCC box elements that share the central AGCCGCC motif, the specificity of the binding of different ERF TFs appears to be rather confusing [49, 69]. *AtRAP2.2* [70] and *AtRAP2.12* [71], two members of the *APETALA2* (AP2)/ethylene-responsive element-binding protein TF family, were found to bind specifically to the ATCTA element. Here, our results show that the *CsPrx73* promoter containing the ATCTA-box and GCC-box is specifically activated by *CsERF7-3* (Fig. 8c and d), consistent with a previous report on the binding activity of *phERF2* [50].

Ethylene, as the first signaling molecule, plays a crucial role in the sensing of waterlogging or hypoxia stress in plants [8]. The Group VII ERFs (ERF-VIIs) have emerged as central regulators in ethylene signal transduction and plant responses to waterlogging [8]. For instance, maize *ZmEREB180* promotes the formation of ARs and the dynamic balance of ROS, and improves the survival rate after long-term waterlogging stress [72]. In contrast, the chrysanthemum ERF VIIIa gene *CmERF4* negatively regulates waterlogging tolerance [73]. In the present study, silencing of *CsERF7-3* results in reduced AR formation (Fig. 9a and b). Conversely, overexpression of *CsERF7-3* promotes AR formation (Fig. 9k). These findings suggest that *CsERF7-3* serves as a positive regulator in waterlogging-triggered AR formation. In addition, our results support the notion of *CsERF7-3* direct interaction with *CsPrx73* both in *vivo* and in *vitro* (Fig. 9d and m).

In conclusion, based on our data, waterlogging activates *CsERF7-3* transcription and operates upstream of *CsPrx73*, triggering its expression. Overexpression of *CsPrx73* has been shown to positively control AR formation in cucumber during waterlogging stress by enhancing ROS scavenging abilities and induction of lignin biosynthesis genes (*Cs4CL*, *CsCAD*, *CsPrx*). In broad terms, the exploration of the *CsERF7-3–CsPrx73* transcriptional cascade enables an investigation of further ERF transcriptional pathways.

## Materials and methods

### Plant material and waterlogging stress treatment

Plant materials YZ026A (waterlogging-tolerant) and YZ106A (waterlogging-sensitive) were employed, with considerably significant differences in AR production under waterlogging conditions. Vermiculite, peat, and perlite (1:3:1, v/v/v) medium was used to sow seeds in a growth chamber with 75% relative humidity, 28°C/18°C light/dark temperature, 16 h/8 h photoperiod, and 280–300 μmol m^−2^ s^−1^ light condition. After 15 days of germination, seedlings at the two-leaf stage were placed in plastic cups for waterlogging, with water up to the tip of the hypocotyl. The control plants were left in the normal growth condition. Hypocotyls of YZ026A and YZ106A were harvested at 12, 24, 48, and 96 h of waterlogging treatment, respectively. For each stress stage, hypocotyls of 15 cucumber seedlings were pooled for each of the three biological replicates and the samples were rapidly placed in liquid nitrogen and stored at −80°C.

### Anatomical observation on hypocotyls

Under waterlogging or normal growth conditions, the basal section of the hypocotyls was harvested and fixed with FAA fixing solution [5% (v/v) formalin, 5% acetic acid, 17% distilled water and 63% ethanol]. Toluidine blue was used to stain the hypocotyl, and the cross-section was promptly cut using an MTH-1 plant slicer (Nippon Medical & Chemical Instruments Co., Ltd, Osaka, Japan). Finally, the formation process of AR primordia was observed under an optical microscope (DM5000 B; Leica Microsystems, Wetzlar, Germany) equipped with a charge-coupled device camera (TrueChrome II; Tucsen Photonics, Fuzhou), and the formation time and morphological characteristics of AR primordia were recorded and photographed at 12, 24, and 48 h of waterlogging.

### RNA sequencing and bioinformatic analysis

TRIzol reagent (Invitrogen, Carlsbad, CA, USA) was used to isolate total RNA. The concentrations and integrity of the total RNA samples were detected using an Agilent 2100 RNA Nano 6000 Assay Kit and a NanoPhotometer^®^ spectrophotometer from Thermo Fisher. Following the instructions provided by Illumina’s TruSeq RNA Sample Prep Kit V2, stranded cDNA libraries were generated from 500 ng of total RNA for every sample. A total of 48 libraries were sequenced with the BGIseq (DNBSEQ T7) sequencing platform at Annoroad (Beijing, China). Under the accession number PRJNA847928, all of the sequencing reads have been uploaded into the NCBI Sequence Read Archive (SRA).

After removing low-quality reads, the clean reads were mapped to the cucumber 9930_v3 reference genome with the BWT algorithm of HISAT2 [74]. By utilizing FPKM, quantitative gene expression levels were identified [75]. Using the criterion of a false discovery rate (FDR) < 0.05 and |log_2_ fold change| > 1.0, the R package DESeq [76] was used to assess DEGs. For each phase, the gene numbers were determined by mapping DEGs to GO terms in the GO database (http://www.geneontology.org/). Using the KEGG database (http://www.genome.jp/kegg), pathway analysis of DEGs was carried out to reveal important pathways.

### Quantitative RT–PCR

In order to verify the accuracy of RNA-seq results, six DEGs were chosen at random from RNA-seq data for the purpose of analyzing their relative expression levels. Hypocotyls were collected independently at 12, 24, 48, and 96 h after white light exposure. The expression levels were assessed using SYBR^®^ Premix Ex Taq™ II (TaKaRa, China) in conjunction with an iQ™ 5 Multicolor Real-Time PCR Detection System (Bio-Rad, USA). The gene expression levels were determined using the 2^−ΔΔCT^ technique [77]. The primers used for the qRT–PCR analysis are documented in Supplementary Data Table S10. Each sample was normalized for mRNA abundance using cucumber *β-actin*, and three duplicates were conducted.

### Weighted gene co-expression network analysis and functional enrichment analysis

The essential modules were screened using weighted gene co-expression network analysis (WGCNA) [47]. Gene expression matrices from transcriptome samples of 48 cucumber hypocotyl samples were first used as input data, followed by screening and filtering of selected genes. A total of 2616 genes with FPKM ≥1, and a variation of FPKM coefficient of variations (cv)  ≥ 0.5 and cv ≤ sd (genes number)/mean (genes number) (where sd represents the standard deviation of the sample, and mean represents the calculated average of the sample) were finally used for WGCNA analysis. pickSoftThreshold and powerEstimate in the WGCNA package were used to calculate the soft thresholding power and estimate the optimal weight value, respectively. The minimum power value (β = 10) when the correlation coefficient reached the plateau (or >0.8) was taken as the weight value, and the change in average gene connectivity under different power values was also counted. The clustered tree was constructed according to the correlation of expression between genes, and the modules were divided according to the dynamic tree cut algorithm with default parameters, where each branch represented one co-expression module. The expression patterns of module genes in each sample are presented as module feature values, and a heat map of sample expression patterns was drawn. Module eigengene analysis was used to identify specific modules significantly associated with waterlogging tolerance, and the corresponding modules were subsequently selected for further study. Cytoscape (version 3.9.1) was used to map the reciprocal networks of genes in the significantly related modules. Genes with high connectivity were screened and used as core genes in the module.

### Enzyme activity determination, lignin determination, and histochemical staining

Commercial assay kits (H2O2-2-Y, SAQ-2-G, POD-2-Y, CAT-2-W, SOD-2-Y, Comin, Suzhou, China; http://www.cominbio.com/) were used to measure ROS content (H_2_O_2_, O^2−^) and POD, CAT, and SOD activities, according to the manufacturer’s instructions.

Hypocotyls were fixed in FAA stationary solution (70% ethanol:formaldehyde:acetic acid, 9:1:1), then dehydrated with graded 50, 70, 90, and 100% ethanol (1 h each). Subsequently, we continued to dehydrate the samples with a mixture of xylene:absolute ethanol (1:3, 1:1, 3:1, v/v) followed by a mixture of xylene:chloroform (9:1, v/v) (every 30 min). The transparent samples were dipped in wax, embedded, sectioned at 8 μm (RM2235, Leica, Germany), deparaffinized, stained, dehydrated, transparent, and mounted. Phloroglucinol solution (5%) was added to the crosssection after dewaxing, and the samples were incubated for 2 min based on the paraffin sections and then sealed using 18% HCl solution. Finally, lignin deposition was observed under an optical microscope (CX31, Olympus, Japan).

Total lignin content was determined with a test kit (MZS-1-G, Suzhou, China; http://www.cominbio.com/) following methods described previously [78]. For each stress stage, hypocotyls from 15 cucumber seedlings were pooled for each biological replicate and at least three were obtained.

### Yeast one-hybrid assays

Y1H assays were conducted using the Matchmaker Gold Yeast One-Hybrid System from TaKaRa, a company based in Japan. A 2000-bp region located before the start codon (ATG) of *CsPrx73* in YZ026A was amplified using PCR. Following Sanger sequencing, the promoter was inserted into a pAbAi vector as bait. The *CsPrx73* promoter was introduced into the yeast strain Y1H-Gold by transfecting it with either the *CsERF7-3*-pGADT7 recombinant vector or the empty pGADT7 vector (used as a negative control) The genetically modified yeast cells were cultivated on a selective medium, SD/−Leu, supplemented with 300 ng/ml of AbA on a plate at a temperature of 28°C for a period of 3–4 days. The primer sequences utilized in Y1H are provided in Supplementary Data Table S10.

### Dual-luciferase assay

The 2000-bp promoter sequence upstream of the ATG starts codon of *CsPrx73* was amplified by PCR and ligated into vector pGreenII0800-LUC to generate p*CsPrx73*::0800-LUC reporter plasmid. The effector plasmid was constructed by inserting the full coding sequence of *CsERF7-3* into the vector pGreenII62-SK. The mixture of p*CsPrx73*::0800-LUC and pGreenII62-SK empty vector was treated as a negative control. As described previously, transformation and infiltration were performed [79]. A Dual-Luciferase Reporter Assay Kit (Vazyme, Nanjing, China) was used to determine the LUC/REN ratios, following the manufacturer’s instructions. A 4600SF Tanon imaging apparatus (Tanon, Shanghai, China) was used to observe the luciferase activity in 5-week-old tobacco leaves. Primers used for transient expression assay are listed in Supplementary Data Table S10.

### Cucumber transformation and phenotypic analysis

For cucumber transformation, the constructed recombinant vector *CsPrx73*-pCAMBIA1301 was transformed into *Agrobacterium* strain EHA105 as defined by Dai *et al*. [80] and Xin *et al*. [81]. The DNA of transgenic lines that detect positive plants was extracted using a rapid plant genomic DNA isolation kit (Tiangen Biotech, Beijing, China). The primer pair 35S-F/R was used to detect positive plants. Additionally, qRT–PCR was performed to identify the relative expression levels. All primers are provided in Supplementary Data Table S10. The phenotype of overexpressing *T*_2_ lines was captured by camera on the 2nd, 4th, and 7th days after waterlogging treatment. The number of ARs in *CsPrx73*-OE and WT were calculated at the 7th day after waterlogging treatment.

### Virus-induced gene silencing

Functional analyses of *CsPrx73* and *CsERF7-3* were conducted using the VIGS system based on the cucumber green mottle mosaic virus, following the method of Liu *et al*. [82]. The *CsPrx73* (from +91 to +390 bp) and *CsERF7-3* (from +211 to +510 bp) coding sequences were amplified from 9930 (Supplementary Data Table S10). The sequences were then inserted into the BamHI site of pV190 vectors to generate pV190-*CsPrx73* and pV190-*CsERF7-3*, respectively. Following the verification of the accuracy of the sequence constructs through Sanger sequencing, the recombinant constructs were introduced into *Agrobacterium tumefaciens* strain GV3101 cells. The *Agrobacterium*-mediated transformation and infection followed the methods described previously [82]. Primers used for VIGS assay are listed in Supplementary Data Table S10.

### Cucumber hairy root transformation

The whole coding sequence of *CsERF7-3* was inserted into the pCAMBIA1300 vector to create the pCAMBIA1300-*CsERF7-3* construct. Following verification of the sequence using Sanger sequencing, the pCAMBIA1300-*CsERF7-3* construct was transformed into *A. rhizogenes* K599 strain. K599 cells containing the pCAMBIA1300-*CsERF7-3* construct or containing the pCAMBIA1300 empty vector were utilized to infect 9930 cotyledons, using a modified technique outlined by Nguyen *et al*. [83]. Primers utilized for hairy root transformation experiment are provided in Supplementary Data Table S10.

## Acknowledgements

We thank the anonymous reviewers for providing valuable comments to improve the manuscript. This work was supported by the National Natural Science Foundation of China (32030093, 32172570, 31972422, and 31801883), the JBGS Project of Seed Industry Revitalization in Jiangsu Province (JBGS[2021]018), and the Jiangsu Agricultural Innovation of New Cultivars (PZCZ201720).

## Author contributions

X.C. and X.X. designed the experiments. J.P. and J.S. performed the RNA-seq analysis and transformation of candidate genes. J.P., W.Y., and Q.H. analyzed the results. J.P. wrote the manuscript. H.S., R.S., X.Q., and X.Y. revised the manuscript. All of the authors have reviewed and approved the final manuscript.

## Data availability

All of the raw RNA-seq reads are deposited in the NCBI Sequence Read Archive (SRA) under the accession number PRJNA847928. All data generated or analyzed that support the findings of this study are included in the main text article and its supplementary files.

## Conflict of interest

The authors declare that they have no competing financial interest.

## Supplementary data

Supplementary data are available at *Horticulture Research* online.

## Supplementary Material

Web_Material_uhae062

## References

[1] Su L , Chen X, Qi X. et al. Involvement of auxin in growth and stress response of cucumber. Vegetable Res. 2022;2:13

[2] Liu K , Harrison MT, Yan H. et al. Silver lining to a climate crisis in multiple prospects for alleviating crop waterlogging under future climates. Nat Commun. 2023;14:1–13 36765112 10.1038/s41467-023-36129-4 PMC9918449

[3] Mira MM , Huang S, Hill RD. et al. Tolerance to excess moisture in soybean is enhanced by over-expression of the *Glycine max* phytoglobin (GmPgb1). Plant Physiol Biochem. 2021;159:322–34 33421908 10.1016/j.plaphy.2020.12.033

[4] Sun L , Ma L, He S. et al. AtrbohD functions downstream of ROP2 and positively regulates waterlogging response in *Arabidopsis*. Plant Signal Behav. 2018;13:e1513300 30188766 10.1080/15592324.2018.1513300 PMC6204828

[5] Zhao N , Li C, Yan Y. et al. Comparative transcriptome analysis of waterlogging-sensitive and waterlogging-tolerant *Chrysanthemum morifolium* cultivars under waterlogging stress and reoxygenation conditions. Int J Mol Sci. 2018;19:1455 29757964 10.3390/ijms19051455 PMC5983694

[6] Qiao D , Zhang Y, Xiong X. et al. Transcriptome analysis on responses of orchardgrass (*Dactylis glomerata* L.) leaves to a short term flooding. Hereditas. 2020;157:20 32418541 10.1186/s41065-020-00134-0 PMC7232843

[7] Steffens B , Rasmussen A. The physiology of adventitious roots. Plant Physiol. 2016;170:603–17 26697895 10.1104/pp.15.01360 PMC4734560

[8] Pan J , Sharif R, Xu X. et al. Mechanisms of waterlogging tolerance in plants: research progress and prospects. Front Plant Sci. 2021;11:627331 33643336 10.3389/fpls.2020.627331 PMC7902513

[9] Pedersen O , Sauter M, Colmer TD. et al. Regulation of root adaptive anatomical and morphological traits during low soil oxygen. New Phytol. 2021;229:42–9 32045027 10.1111/nph.16375

[10] Xu X , Chen M, Ji J. et al. Comparative RNA-seq based transcriptome profiling of waterlogging response in cucumber hypocotyls reveals novel insights into the *de novo* adventitious root primordia initiation. BMC Plant Biol. 2017;17:129 28747176 10.1186/s12870-017-1081-8 PMC5530484

[11] Eysholdt Derzso E , Sauter M. Hypoxia and the group VII ethylene response transcription factor HRE2 promote adventitious root elongation in *Arabidopsis*. Plant Biol. 2019;21:103–8 29996004 10.1111/plb.12873 PMC6585952

[12] Kramer PJ . Causes of injury to plants resulting from flooding of the soil. Plant Physiol. 1951;26:722–36 16654407 10.1104/pp.26.4.722 PMC437542

[13] Sartoris GB , Belcher BA. The effect of flooding on flowering and survival of sugar cane. Sugar. 1949;44:36–9

[14] Lin C , Ogorek LLP, Pedersen O. et al. Oxygen in the air and oxygen dissolved in the floodwater both sustain growth of aquatic adventitious roots in rice. J Exp Bot. 2021;72:1879–90 33206163 10.1093/jxb/eraa542

[15] Mano Y , Omori F, Loaisiga CH. et al. QTL mapping of above-ground adventitious roots during flooding in maize x teosinte “*Zea nicaraguensis*” backcross population. Plant Root. 2009;3:3–9

[16] Wu J , Cheng J, Xu C. et al. AUREA maintains the balance between chlorophyll synthesis and adventitious root formation in tomato. Hortic Res. 2020;7:166 33082972 10.1038/s41438-020-00386-x PMC7527990

[17] Dawood T , Yang X, Visser EJ. et al. A co-opted hormonal cascade activates dormant adventitious root primordia upon flooding in *Solanum dulcamara*. Plant Physiol. 2016;170:2351–64 26850278 10.1104/pp.15.00773 PMC4825138

[18] Qi XH , Li QQ, Ma XT. et al. Waterlogging-induced adventitious root formation in cucumber is regulated by ethylene and auxin through reactive oxygen species signalling. Plant Cell Environ. 2019;42:1458–70 30556134 10.1111/pce.13504

[19] Lin C , Sauter M. Polar auxin transport determines adventitious root emergence and growth in rice. Front Plant Sci. 2019;10:444 31024605 10.3389/fpls.2019.00444 PMC6465631

[20] Vidoz ML , Loreti E, Mensuali A. et al. Hormonal interplay during adventitious root formation in flooded tomato plants. Plant J. 2010;63:551–62 20497380 10.1111/j.1365-313X.2010.04262.x

[21] Arbona V , Gomez CA. Hormonal modulation of citrus responses to flooding. J Plant Growth Regul. 2008;27:241–50

[22] Xu X , Ji J, Ma X. et al. Comparative proteomic analysis provides insight into the key proteins involved in cucumber (*Cucumis sativus* L.) adventitious root emergence under waterlogging stress. Front Plant Sci. 2016;7:1515 27790230 10.3389/fpls.2016.01515 PMC5062059

[23] Shigeto J , Tsutsumi Y. Diverse functions and reactions of class III peroxidases. New Phytol. 2016;209:1395–402 26542837 10.1111/nph.13738

[24] Tognolli M , Penel C, Greppin H. et al. Analysis and expression of the class III peroxidase large gene family in *Arabidopsis thaliana*. Gene. 2002;288:129–38 12034502 10.1016/s0378-1119(02)00465-1

[25] Passardi F , Longet D, Penel C. et al. The class III peroxidase multigenic family in rice and its evolution in land plants. Phytochemistry. 2004;65:1879–93 15279994 10.1016/j.phytochem.2004.06.023

[26] Ren L , Liu Y, Liu H. et al. Subcellular relocalization and positive selection play key roles in the retention of duplicate genes of *Populus* class III peroxidase family. Plant Cell. 2014;26:2404–19 24934172 10.1105/tpc.114.124750 PMC4114941

[27] Cao Y , Han Y, Meng D. et al. Structural, evolutionary, and functional analysis of the class III peroxidase gene family in Chinese pear (*Pyrus bretschneideri*). Front Plant Sci. 2016;7:1874 28018406 10.3389/fpls.2016.01874 PMC5145892

[28] Hiraga S , Sasaki K, Ito H. et al. A large family of class III plant peroxidases. Plant Cell Physiol. 2001;42:462–8 11382811 10.1093/pcp/pce061

[29] Almagro L , Ros LVG, Belchi Navarro S. et al. Class III peroxidases in plant defence reactions. J Exp Bot. 2009;60:377–90 19073963 10.1093/jxb/ern277

[30] Kunieda T , Shimada T, Kondo M. et al. Spatiotemporal secretion of *PEROXIDASE36* is required for seed coat mucilage extrusion in *Arabidopsis*. Plant Cell. 2013;25:1355–67 23572548 10.1105/tpc.113.110072 PMC3663273

[31] Hou X , Lu Z, Hong K. et al. The class III peroxidase gene family is involved in ascorbic acid induced delay of internal browning in pineapple. Front Plant Sci. 2022;13:953623 35991401 10.3389/fpls.2022.953623 PMC9382127

[32] Liszkay A , van der Zalm E, Schopfer P. Production of reactive oxygen intermediates (O^2−^, H_2_O_2_, and OH) by maize roots and their role in wall loosening and elongation growth. Plant Physiol. 2004;136:3114–23 15466236 10.1104/pp.104.044784 PMC523372

[33] McInnis SM , Desikan R, Hancock JT. et al. Production of reactive oxygen species and reactive nitrogen species by angiosperm stigmas and pollen: potential signalling crosstalk? New Phytol. 2006;172:221–8 16995910 10.1111/j.1469-8137.2006.01875.x

[34] Mangano S , Denita-Juarez SP, Choi HS. et al. Molecular link between auxin and ROS-mediated polar growth. Proc Natl Acad Sci USA. 2017;114:5289–94 28461488 10.1073/pnas.1701536114 PMC5441824

[35] Francoz E , Ranocha P, Nguyen Kim H. et al. Roles of cell wall peroxidases in plant development. Phytochemistry. 2015;112:15–21 25109234 10.1016/j.phytochem.2014.07.020

[36] Raggi S , Ferrarini A, Delledonne M. et al. The *Arabidopsis* class III peroxidase *AtPRX71* negatively regulates growth under physiological conditions and in response to cell wall damage. Plant Physiol. 2015;169:2513–25 26468518 10.1104/pp.15.01464 PMC4677920

[37] Kim BH , Kim SY, Nam KH. Genes encoding plant-specific class III peroxidases are responsible for increased cold tolerance of the brassinosteroid-insensitive 1 mutant. Mol Cells. 2012;34:539–48 23180292 10.1007/s10059-012-0230-z PMC3887832

[38] Kumar S , Jaggi M, Sinha AK. Ectopic overexpression of vacuolar and apoplastic *Catharanthus roseus* peroxidases confers differential tolerance to salt and dehydration stress in transgenic tobacco. Protoplasma. 2012;249:423–32 21643888 10.1007/s00709-011-0294-1

[39] Chiang H , Lo J, Yeh K. Genes associated with heavy metal tolerance and accumulation in Zn/Cd hyperaccumulator *Arabidopsis halleri*: a genomic survey with cDNA microarray. Environ Sci Technol. 2006;40:6792–8 17144312 10.1021/es061432y

[40] Jouili H , Bouazizi H, Rossignol M. et al. Partial purification and characterization of a copper-induced anionic peroxidase of sunflower roots. Plant Physiol Biochem. 2008;46:760–7 18586509 10.1016/j.plaphy.2008.04.006

[41] Wu Y , Yang Z, How J. et al. Overexpression of a peroxidase gene (*AtPrx64*) of *Arabidopsis thaliana* in tobacco improves plant's tolerance to aluminum stress. Plant Mol Biol. 2017;95:157–68 28815457 10.1007/s11103-017-0644-2

[42] Kidwai M , Dhar YV, Gautam N. et al. *Oryza sativa* class III peroxidase (*OsPRX38*) overexpression in *Arabidopsis thaliana* reduces arsenic accumulation due to apoplastic lignification. J Hazard Mater. 2019;362:383–93 30245406 10.1016/j.jhazmat.2018.09.029

[43] Daudi A , Cheng Z, O'Brien JA. et al. The apoplastic oxidative burst peroxidase in *Arabidopsis* is a major component of pattern-triggered immunity. Plant Cell. 2012;24:275–87 22247251 10.1105/tpc.111.093039 PMC3289579

[44] Lorrai R , Francocci F, Gully K. et al. Impaired cuticle functionality and robust resistance to *Botrytis cinerea* in *Arabidopsis thaliana* plants with altered homogalacturonan integrity are dependent on the class III peroxidase *AtPRX71*. Front Plant Sci. 2021;12:696955 34484262 10.3389/fpls.2021.696955 PMC8415794

[45] Pan J , Tu J, Sharif R. et al. Study of JASMONATE ZIM-domain gene family to waterlogging stress in *Cucumis sativus* L. Vegetable Res. 2021;1:1–12

[46] Tu J . Germplasm identification of waterlogging tolerant and QTL mapping for waterlogging tolerance in cucumber. MA Thesis. Yangzhou University; 2022: (in Chinese)

[47] Langfelder P , Horvath S. WGCNA: an R package for weighted correlation network analysis. BMC Bioinformatics. 2008;9:559 19114008 10.1186/1471-2105-9-559 PMC2631488

[48] Shannon P , Markiel A, Ozier O. et al. Cytoscape: a software environment for integrated models of biomolecular interaction networks. Genome Res. 2003;13:2498–504 14597658 10.1101/gr.1239303 PMC403769

[49] Ohme Takagi M , Shinshi H. Ethylene-inducible DNA binding proteins that interact with an ethylene-responsive element. Plant Cell. 1995;7:173–82 7756828 10.1105/tpc.7.2.173 PMC160773

[50] Yin D , Sun D, Han Z. et al. *PhERF2*, an ethylene-responsive element binding factor, plays an essential role in waterlogging tolerance of petunia. Hortic Res. 2019;6:83 31645944 10.1038/s41438-019-0165-z PMC6804856

[51] Mustroph A . Improving flooding tolerance of crop plants. Agronomy. 2018;8:160

[52] Manik SMN , Pengilley G, Dean G. et al. Soil and crop management practices to minimize the impact of waterlogging on crop productivity. Front Plant Sci. 2019;10:140 30809241 10.3389/fpls.2019.00140 PMC6379354

[53] Xu X , Ji J, Xu Q. et al. The major-effect quantitative trait locus CsARN6.1 encodes an AAA ATPase domain-containing protein that is associated with waterlogging stress tolerance by promoting adventitious root formation. Plant J. 2018;93:917–30 29315927 10.1111/tpj.13819

[54] Qi X , Li Q, Shen J. et al. Sugar enhances waterlogging-induced adventitious root formation in cucumber by promoting auxin transport and signalling. Plant Cell Environ. 2020;43:1545–57 32020637 10.1111/pce.13738

[55] Cao B , Li N, Xu K. Crosstalk of phenylpropanoid biosynthesis with hormone signaling in Chinese cabbage is key to counteracting salt stress. Environ Exp Bot. 2020;179:104209

[56] Geng D , Shen X, Xie Y. et al. Regulation of phenylpropanoid biosynthesis by MdMYB88 and MdMYB124 contributes to pathogen and drought resistance in apple. Hortic Res. 2020;7:102 32637130 10.1038/s41438-020-0324-2 PMC7327078

[57] Vanholme R , Demedts B, Morreel K. et al. Lignin biosynthesis and structure. Plant Physiol. 2010;153:895–905 20472751 10.1104/pp.110.155119 PMC2899938

[58] Hasanuzzaman M , Bhuyan MHMB, Anee TI. et al. Regulation of ascorbate-glutathione pathway in mitigating oxidative damage in plants under abiotic stress. Antioxidants. 2019;8:384 31505852 10.3390/antiox8090384 PMC6770940

[59] Sohail H , Noor I, Nawaz MA. et al. Genome-wide identification of plasma-membrane intrinsic proteins in pumpkin and functional characterization *CmoPIP1-4* under salinity stress. Environ Exp Bot. 2022;202:104995

[60] Dumanovic J , Nepovimova E, Natic M. et al. The significance of reactive oxygen species and antioxidant defense system in plants: a concise overview. Front Plant Sci. 2021;11:552969 33488637 10.3389/fpls.2020.552969 PMC7815643

[61] Hasanuzzaman M , Bhuyan MHMB, Zulfiqar F. et al. Reactive oxygen species and antioxidant defense in plants under abiotic stress: revisiting the crucial role of a universal defense regulator. Antioxidants. 2020;9:681 32751256 10.3390/antiox9080681 PMC7465626

[62] Li C , Su J, Zhao N. et al. *CmERF5-CmRAP2.3* transcriptional cascade positively regulates waterlogging tolerance in *Chrysanthemum morifolium*. Plant Biotechnol J. 2023;21:270–82 36200911 10.1111/pbi.13940 PMC9884023

[63] Bhatt I , Tripathi BN. Plant peroxiredoxins: catalytic mechanisms, functional significance and future perspectives. Biotechnol Adv. 2011;29:850–9 21777667 10.1016/j.biotechadv.2011.07.002

[64] Liu H , Dong SY, Li M. et al. The class III peroxidase gene *OsPrx30*, transcriptionally modulated by the AT-hook protein OsATH1, mediates rice bacterial blight-induced ROS accumulation. J Integr Plant Biol. 2021;63:393–408 33241917 10.1111/jipb.13040

[65] Jin T , Sun Y, Zhao R. et al. Overexpression of peroxidase gene *GsPRX9* confers salt tolerance in soybean. Int J Mol Sci. 2019;20:3745 31370221 10.3390/ijms20153745 PMC6695911

[66] Su P , Yan J, Li W. et al. A member of wheat class III peroxidase gene family, *TaPRX-2A*, enhanced the tolerance of salt stress. BMC Plant Biol. 2020;20:392 32847515 10.1186/s12870-020-02602-1 PMC7449071

[67] Zhang H , Wang Z, Li X. et al. The *IbBBX24*-*IbTOE3*-*IbPRX17* module enhances abiotic stress tolerance by scavenging reactive oxygen species in sweet potato. New Phytol. 2022;233:1133–52 34773641 10.1111/nph.17860

[68] Marzol E , Borassi C, Carignani Sardoy M. et al. Class III peroxidases *PRX01*, *PRX44*, and *PRX73* control root hair growth in *Arabidopsis thaliana*. Int J Mol Sci. 2022;23:5375 35628189 10.3390/ijms23105375 PMC9141322

[69] Hao D , Ohme Takagi M, Sarai A. Unique mode of GCC box recognition by the DNA-binding domain of ethylene-responsive element-binding factor (ERF domain) in plant. J Biol Chem. 1998;273:26857–61 9756931 10.1074/jbc.273.41.26857

[70] Welsch R , Maass D, Voegel T. et al. Transcription factor RAP2.2 and its interacting partner SINAT2: stable elements in the carotenogenesis of *Arabidopsis* leaves. Plant Physiol. 2007;145:1073–85 17873090 10.1104/pp.107.104828 PMC2048778

[71] Licausi F , Kosmacz M, Weits DA. et al. Oxygen sensing in plants is mediated by an N-end rule pathway for protein destabilization. Nature. 2011;479:419–22 22020282 10.1038/nature10536

[72] Yu F , Liang K, Fang T. et al. A group VII ethylene response factor gene, ZmEREB180, coordinates waterlogging tolerance in maize seedlings. Plant Biotechnol J. 2019;17:2286–98 31033158 10.1111/pbi.13140 PMC6835127

[73] Li C , Wang L, Su J. et al. A group VIIIa ethylene-responsive factor, *CmERF4*, negatively regulates waterlogging tolerance in chrysanthemum. J Exp Bot. 2023;75:1479–92 10.1093/jxb/erad451 37952115

[74] Siren J , Valimaki N, Makinen V. Indexing graphs for path queries with applications in genome research. IEEE/ACM Trans Comput Biol Bioinform. 2014;11:375–88 26355784 10.1109/TCBB.2013.2297101

[75] Trapnell C , Williams BA, Pertea G. et al. Transcript assembly and quantification by RNA-seq reveals unannotated transcripts and isoform switching during cell differentiation. Nat Biotechnol. 2010;28:511–5 20436464 10.1038/nbt.1621 PMC3146043

[76] Wang L , Feng Z, Wang X. et al. DEGseq: an R package for identifying differentially expressed genes from RNA-seq data. Bioinformatics. 2010;26:136–8 19855105 10.1093/bioinformatics/btp612

[77] Livak KJ , Schmittgen TD. Analysis of relative gene expression data using real-time quantitative PCR and the 2^−ΔΔCT^ method. Methods. 2001;25:402–8 11846609 10.1006/meth.2001.1262

[78] Zhao D , Luan Y, Shi W. et al. Melatonin enhances stem strength by increasing lignin content and secondary cell wall thickness in herbaceous peony. J Exp Bot. 2022;73:5974–91 35436332 10.1093/jxb/erac165

[79] Wang Y , Zhou L, Wang Y. et al. *CmMYB9a* activates floral coloration by positively regulating anthocyanin biosynthesis in chrysanthemum. Plant Mol Biol. 2022;108:51–63 34714494 10.1007/s11103-021-01206-z

[80] Dai HB , Zhu ZH, Wang ZG. et al. Galactinol synthase 1 improves cucumber performance under cold stress by enhancing assimilate translocation. Hortic Res. 2022;9:uhab063 35048123 10.1093/hr/uhab063 PMC9015895

[81] Xin T , Tian H, Ma Y. et al. Targeted creation of new mutants with compact plant architecture using CRISPR/Cas9 genome editing by an optimized genetic transformation procedure in cucurbit plants. Hortic Res. 2022;9:uhab086 35048122 10.1093/hr/uhab086 PMC9016859

[82] Liu M , Liang Z, Aranda MA. et al. A cucumber green mottle mosaic virus vector for virus-induced gene silencing in cucurbit plants. Plant Methods. 2020;16:9 32025236 10.1186/s13007-020-0560-3 PMC6996188

[83] Nguyen DV , Hoang TT, Le NT. et al. An efficient hairy root system for validation of plant transformation vector and CRISPR/Cas construct activities in cucumber (*Cucumis sativus* L.). Front Plant Sci. 2022;12:770062 35222448 10.3389/fpls.2021.770062 PMC8874011

