# Supplementary material for: RNA-seq-based comparative transcriptome analysis reveals the role of CsPrx73 in waterlogging-triggered adventitious root formation in cucumber
Source: Hortic Res. 2024 Feb 28;11(4):uhae062. doi: 10.1093/hr/uhae062 (PMC11040206; doi:10.1093/hr/uhae062)
Supplement: Web_Material_uhae062 [file web_material_uhae062.zip › Figure S6.pdf]

ATGCATGTCGATTTGTAGGGTTGAGAATATATCATTGAATAAAAACCTACAAAACAGGTTTTAGTACAAACTAAAAGCAGTTTAAAATATATTAGTTGAAGT
GATGACAAAAAGGGTTCAAGTGCTTGTAAATTTATTGACATTTTATACATGCAGATCTAAATCGAAATATTTGTCATAAATTAACAACAATAATTGTTACTTG
TAAACTAAAATAATCTTGGGTTTGTCCCAATCCCCCACCCAATTGATTTAAGAAAAATATCCAAATCTCAGGGCCATTAATTTTTTTAAGCACATAGAAAA
GTTTTAAGAAATTATGGATGATGAAACAATTATTAAAATTTAATTATACTTTAATTCAAACGAGTCAGGAATATCTATCCTATTAATATATATCTCTTTCATTG
TTAAATGGTAATACAATTCATATTTATCGTTTAAATTGCATGCTTAAACACCATACAATGCAACAATTAGTAGTTAATCATTTTACTTTTAAATTGTGATGTTT
TATATCTACAATGATTTAAATTGTACATTTAAATTGTGATGTTTTATATCTACAATGATTTAAATTACACATGTGCACAAAAAATAACTTATTTGTTGTAACAA
AAATTTATAAACATTCTAAGAACAAAATAAAATTGCAAGGATTCTCCATTATCCATAAAATTACATATAGATTGTAGATACTAATATAGAGCTATTGTCTCAA
TATATGATGTACACATGTTTATGTCTATTTGTAAAGTTTAAATGAAGGTAAATGAGACTAATCTTACAATATCTTTGTATAATATTAAACACATGTGTGAGCC
AACAAAGGAATGTTATGCATGTTTATTGGTACTTTTCTCTCTCTCTCTCTCGTTTTGTTGGAGGTGGAAGTTTTCAAAGAGACAGAATTTTACGTTTTAATA
TACTTTTTATATTCAAATTTAAAATTTTACTCGGAAGAGGAAAATAAACTTCAAAATTAATCATTTAACTTTTCGAAATGAACTTTTGATATAAAATAACATAA
GGTGTCAATTTCCCCTCCCAAATTTCTTCCTTACATCGTGGTTAGTGAACAAATTAAAACAAGAAAGAAAATGAGTTACAAATGTAAAATTAACTGAATTTA
AATGGTAAACCTTTTACTTATAGTATTCCCAATAAATATAGTGCAATATCATTTTTCATAAAAAATGATTTTTTTTTTTTTCATATGACAAATTGTGACTAATAA
TTTGATGTGAGGAAGGGGAGGTAGATGTGTAAATTTGGGTAAGAAAAGGAAGTTAGAAGCAGCGTGGGTGATGGATTGATTGGTGTCCAAAATTTCAAC
TTTTGGGATGAGCCCATCGGCCATCACCCCACCCACCTATTTACCTACACCTTATACTACCACTACACAATAGCAAAAGCCTTAACTTGGATGCTAAGTT
TTTGTTATACCCTTTCCCCCTTTACCTTTGCCATTGGACCAAACTACTTTTTTTTCTTTTTTTCTCTTTTATATCTCTTTGAATATTCTAATTCCTTTCAAGTTT
CAATCATGCCCTAATTTTCCTCTCCATTTGTACTTATTATTGTGGTACATAACTACAGTCCATTGGCGGCCATTGCCCAACACTATTATAAAGCCTCCATT
TCTGAATATTAATCCACCCCTCTCCTTTATCCAATCCCTATTTCTATTCAATCTCAATCATGCATATACATATGTGTTTGTATTTTTTTAAAATTTTCCTAATA
ATACCCATTTGATTTTCCGTGAGTTATTACTTGTGTCACTAAGCATATTCCAAGTATAGAATTTTCCACTCTAATTATAATTAATTTTTTTTTAAATGAAAAAA
ATGGAAAAAACGAAAAGCAAATTGATTGGGAATTTTGATCCAACTGGCTAATTAGCCGCCTCCACTCTTATATATTCCTACTGCTCCCCCATTAATGCTG
TGCTCATATCCTTCTTCCCCTCCTCCCCCAATTTTTATTAATTTGCTAAA
