# Supplementary figures and images for: RNA-seq-based comparative transcriptome analysis reveals the role of CsPrx73 in waterlogging-triggered adventitious root formation in cucumber
Source: Hortic Res. 2024 Feb 28;11(4):uhae062. doi: 10.1093/hr/uhae062 (PMC11040206; doi:10.1093/hr/uhae062)

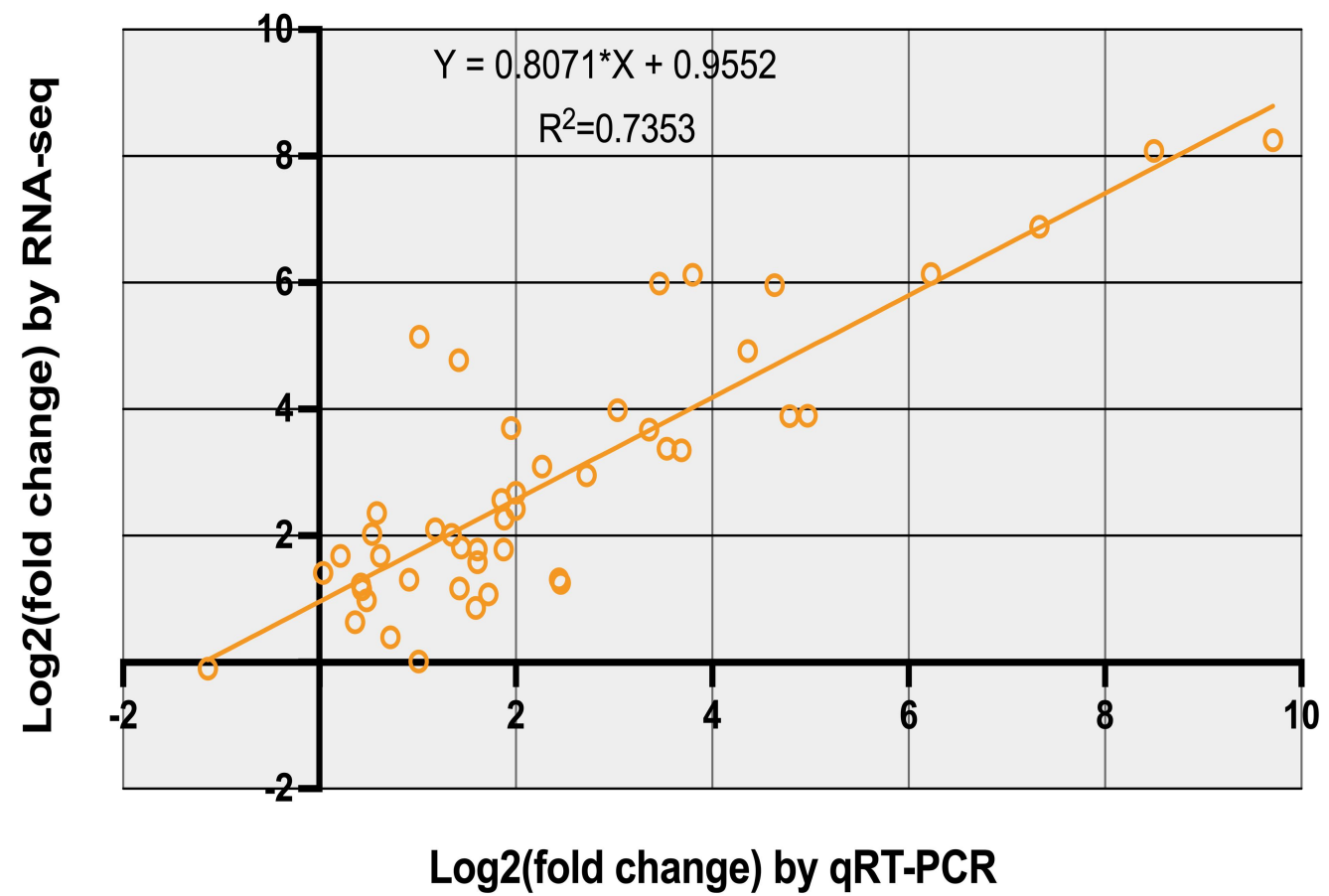

Supplement: Web_Material_uhae062 [file web_material_uhae062.zip › Figure S1.pdf]

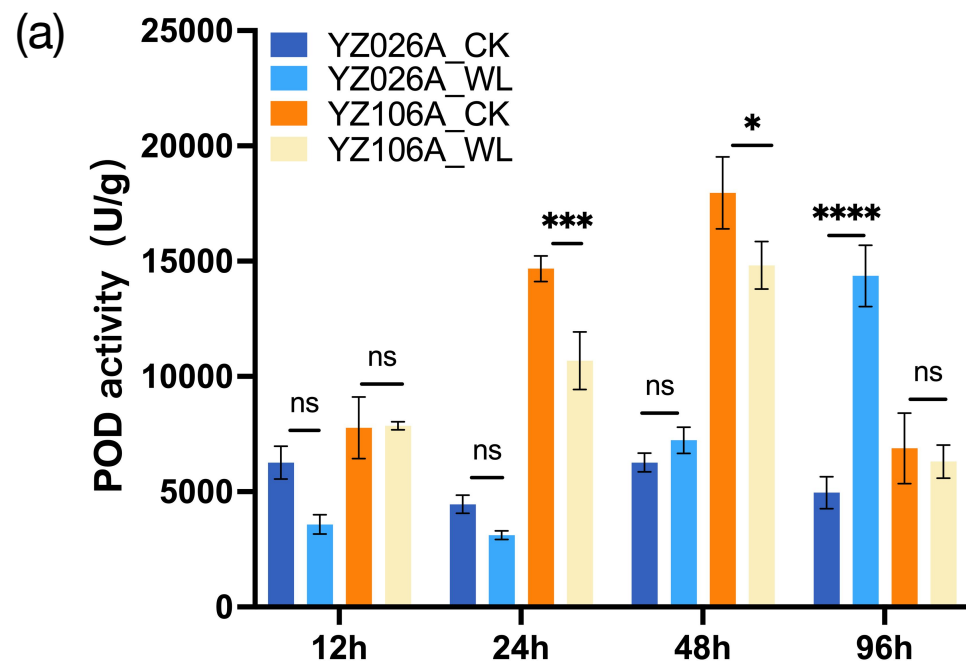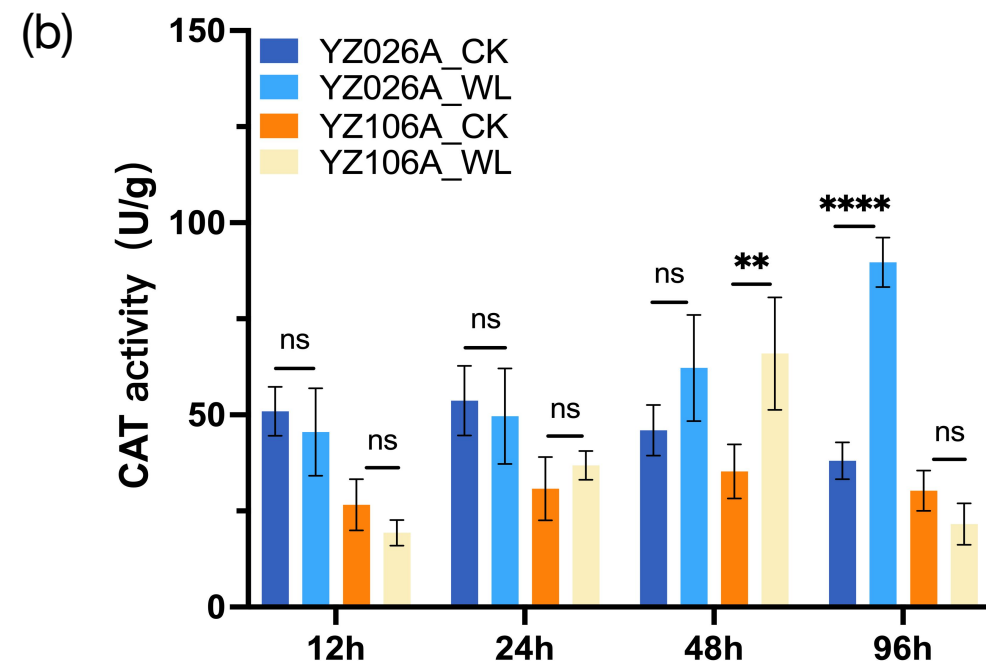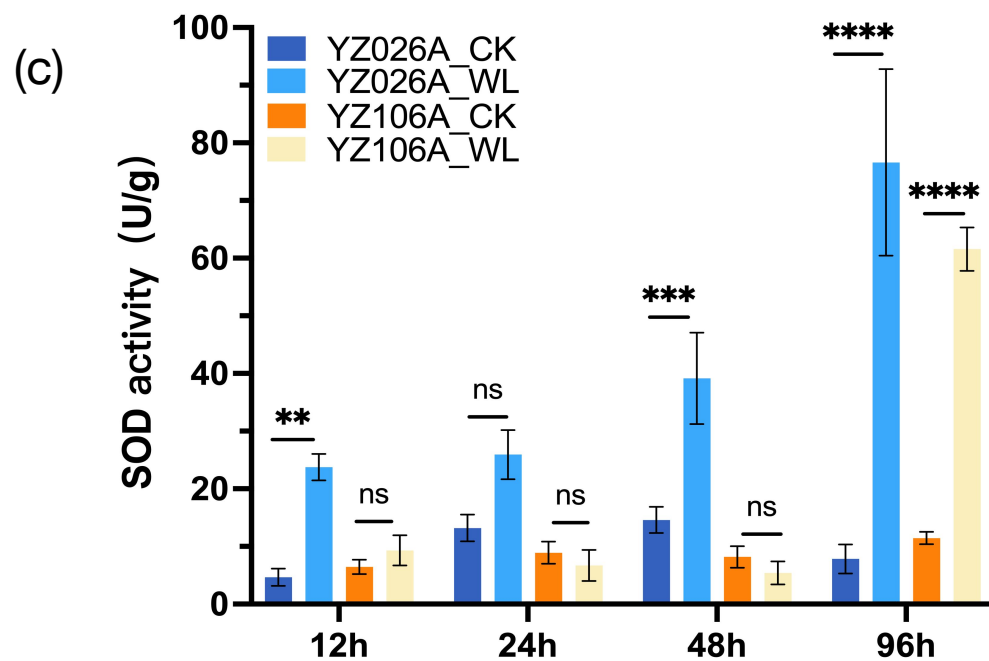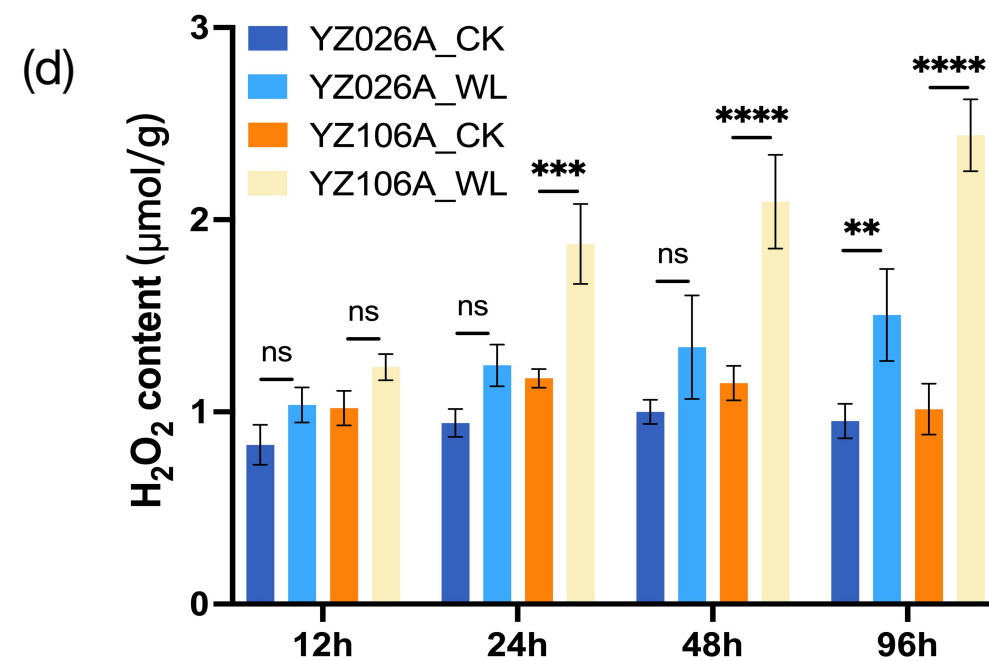

Supplement: Web_Material_uhae062 [file web_material_uhae062.zip › Figure S2.pdf]

## Top 20 of KEGG Enrichment

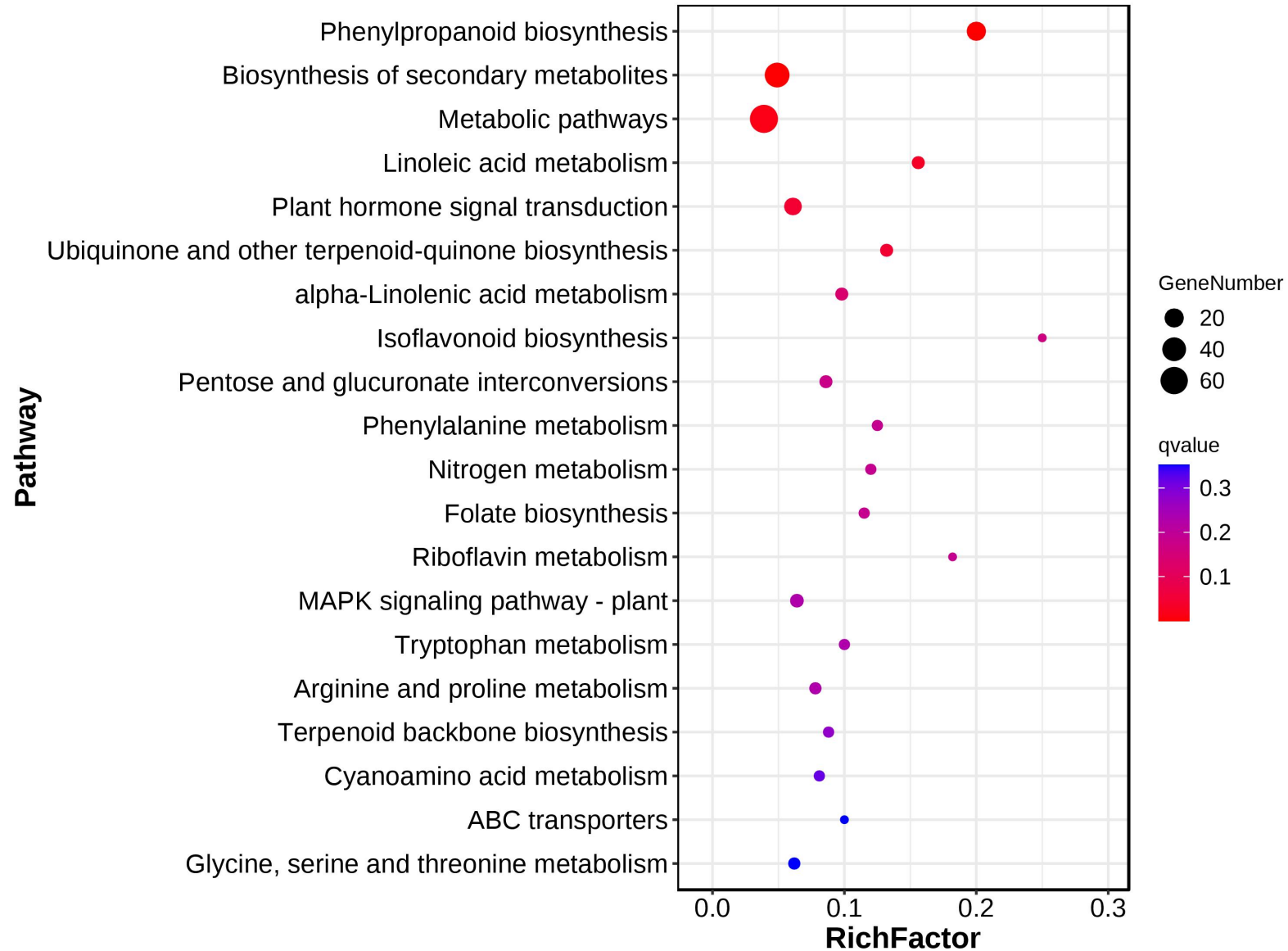

Supplement: Web_Material_uhae062 [file web_material_uhae062.zip › Figure S3.pdf]

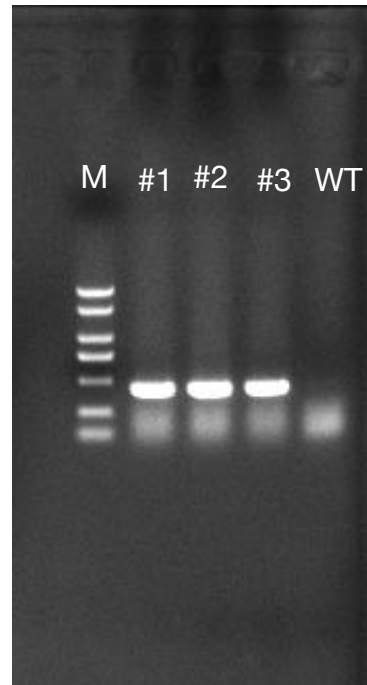

Supplement: Web_Material_uhae062 [file web_material_uhae062.zip › Figure S4.pdf]

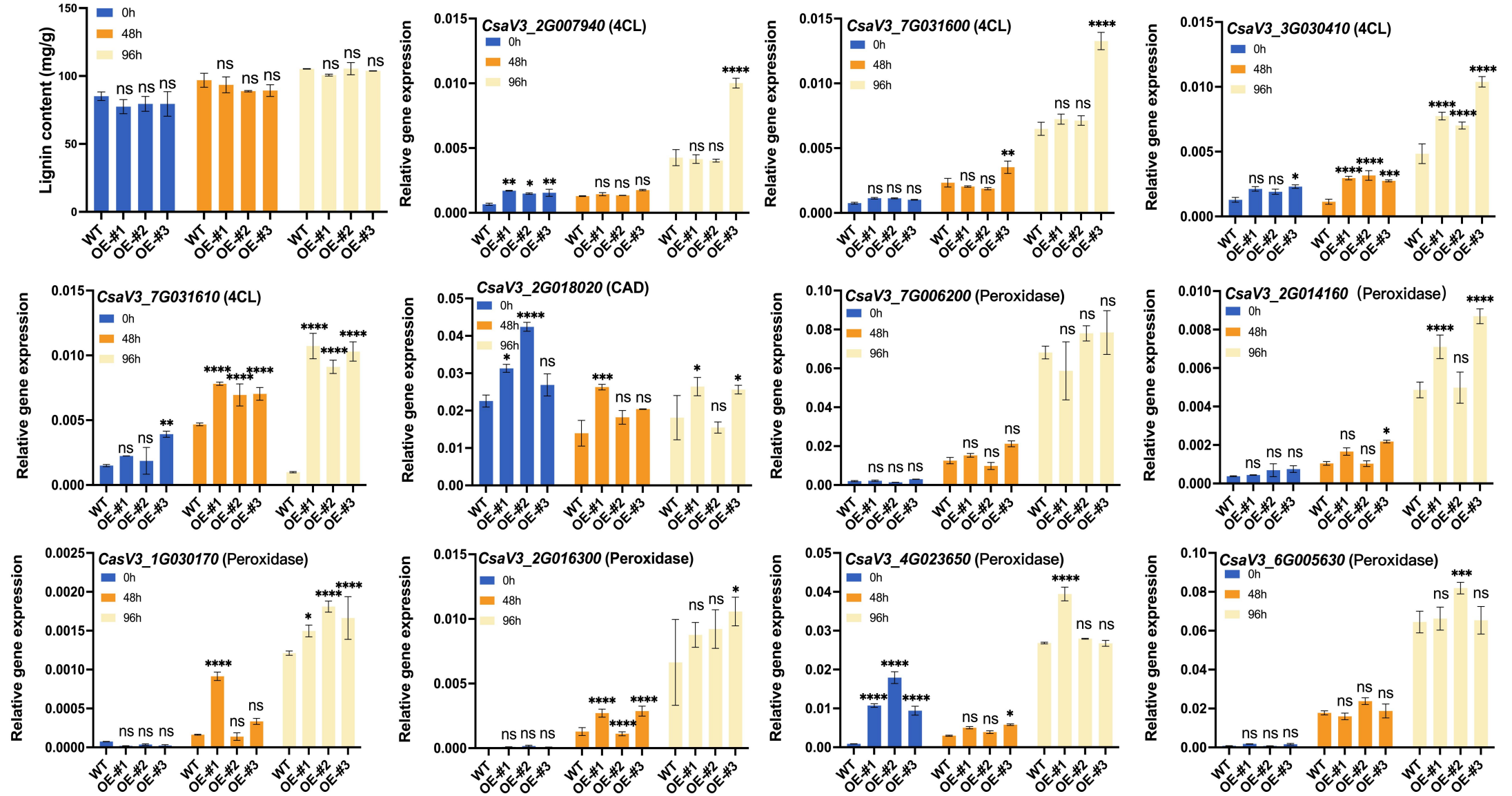

Supplement: Web_Material_uhae062 [file web_material_uhae062.zip › Figure S5.pdf]
